# Unraveling the Food Matrix Components Underlying Glycemic Response in Yellow Popcorn Hybrids (*Zea mays* var. ‘Everta’)

**DOI:** 10.3390/foods15183333

**Published:** 2026-09-20

**Authors:** Emiko Daniela González-Nimi, Janet A. Gutiérrez-Uribe, Benjamín Vázquez-Rodríguez, Marilena Antunes-Ricardo, Ricardo Ernesto Preciado-Ortiz, Anayansi Escalante-Aburto

**Affiliations:** 1 Tecnologico de Monterrey, School of Engineering and Sciences, Av. Eugenio Garza Sada 2501, Monterrey 64849, Nuevo León, Mexico; a01324618@tec.mx (E.D.G.-N.); bv@tec.mx (B.V.-R.); marilena.antunes@tec.mx (M.A.-R.); 2 Tecnologico de Monterrey, Institute for Obesity Research, Av. Eugenio Garza Sada 2501, Monterrey 64849, Nuevo León, Mexico; 3 Programa de Mejoramiento Genético de Maíz, Campo Experimental del Bajío, Instituto Nacional de Investigaciones Forestales, Agrícolas y Pecuarias (INIFAP), Carretera Celaya-San Miguel de Allende km 6.5, Celaya 38110, Guanajuato, Mexico; repreciado@yahoo.com

**Keywords:** popcorn (*Zea mays* ‘Everta’), particle size, *in vitro* glycemic index, dietary fiber, phytosterols, phenolics, antioxidant activity

## Abstract

Most popcorn studies have focused on individual components, while the interactions among phytochemicals, particle size, and glycemic response remain poorly understood. This study evaluated five popcorn hybrids and one commercial sample, expanded by hot air and separated into fine and coarse fractions, to identify matrix components associated with estimated glycemic index (eGI). *In vitro* eGI, dietary fiber, antioxidant activity, phenolics, hydroxycinnamic acid amides (HCAA), and phytosterols were evaluated. Hybrids showed low eGI values (45.6–48.3) compared with commercial popcorn, while Popcorn 2 exhibited the highest soluble dietary fiber and lowest eGI. Significant differences in phenolics, HCAA, and phytosterols were observed among samples and particle-size fractions. Free and bound phenolics and phytosterols were predominantly concentrated in fine fractions, whereas HCAA were generally higher in coarse fractions. Bound p-coumaric acid (469 μg/g), ferulic acid (6264 μg/g), and HCAA (up to 1304 μg/g) were higher in fine fractions. β-Sitosterol reached 94.3 μg/g in the fine fraction of Popcorn 5. Free phenolics correlated positively with eGI, suggesting that individual phytochemicals do not independently explain glycemic differences. The eGI variation among popcorn hybrids appears to reflect the combined influence of dietary fiber, particle size, and overall matrix composition rather than individual components.

## 1. Introduction

Over 25 kernel types are used in breeding programs to improve agronomic performance, crop yield, popping quality, expansion characteristics, and nutritional traits [1]. Among these, popcorn has attracted considerable attention as a whole-grain snack because it retains the bran and germ, providing dietary fiber, protein, vitamins, minerals, and a wide range of phytochemicals with antioxidant properties [2]. Popcorn is regarded as a low-energy-density snack and may represent a healthier alternative to many refined snack foods, as its whole-grain structure and nutrient composition have been associated with improved satiety and potential benefits for metabolic health [3,4].

In this sense, the metabolic effects of consuming high-carbohydrate foods directly affect the glycemic index (GI). It reflects the blood glucose response after carbohydrate-rich meals. Instead of being based only on starch content, the GI is influenced by the structural features of the food matrix, which regulate starch accessibility and enzymatic hydrolysis during digestion [5,6]. On the other hand, dietary fiber, resistant starch, proteins, lipids, and phenolic compounds can slow starch digestion by forming physical barriers, establishing molecular interactions with starch, or inhibiting digestive enzymes such as α-amylase and α-glucosidase, thereby lowering post-meal blood sugar levels [7].

The glycemic index of popcorn snacks has been analyzed by Çetin-Babaoğlu et al. [8]. However, little is known about how differences in food matrix composition and particle size influence the glycemic response, particularly their relationship to the phenolic profile. Moreover, most studies on these products have focused on evaluating the contribution of isolated macronutrients rather than integrating the whole matrix to identify the components responsible for glycemic modulation. In addition, comparative information across popcorn genotypes and on changes in matrix composition associated with processing remains limited, making it difficult to determine whether differences in phytochemical and fiber profiles among hybrids are related to their glycemic response. This information is particularly valuable for grain breeders.

As previously described, particle size represents an important factor when evaluating the nutritional value of foods [9]. During mastication, popcorn generates heterogeneous particle-size fractions with distinct anatomical parts of the grain and, hence, compositional characteristics. A study conducted by Escalante-Aburto et al. [10] focused on the evaluation of the *in vitro* simulated digestion of the whole fraction of pigmented popcorn expanded by microwave. However, they do not differentiate between the coarse and fine fractions, which could affect digestibility and functional characteristics. These fine and coarse fractions differ significantly in their endosperm, germ, and pericarp tissue content, leading to differences in dietary fiber, starch, and phenolic compound content [6,11,12]. Particularly, the coarse fraction of hot air-popped popcorn contains more bound phenolics and resistant starch than the fine fraction [13]. Nevertheless, the relationships among genotype, particle size, phytochemical composition, and starch digestion in popcorn remain poorly understood. A comparative evaluation of grain and popcorn, along with their fine and coarse fractions, may therefore provide insights into how variations in the food matrix influence glycemic response. Therefore, examining both fractions is essential to fully understand the popcorn matrix and to explore the effect of genotype and processing on phytochemicals and starch digestion, and, consequently, the glycemic index of this snack.

This study aimed to analyze the food matrix and phytochemical composition of five yellow popcorn hybrids (*Zea mays* var. ‘Everta’) and their influence on *in vitro* glycemic response. Dietary fiber, phenolic compounds, phytosterols, and antioxidant activity were characterized in fine and coarse fractions of popped kernels to identify key matrix components associated with a reduced glycemic response.

## 2. Materials and Methods

### 2.1. Biological Material

Five yellow popcorn hybrids (*Zea mays* var. ‘Everta’), developed in 2024 at the experimental fields of the “Instituto Nacional de Investigaciones Forestales, Agrícolas y Pecuarias” (INIFAP), were utilized, with a commercial popcorn used as a control (C). The grains were sifted through a 149 µm US sieve (mesh No. 100) to eliminate foreign material and stored at 4 °C in sealed polyethylene bags until use.

### 2.2. Popping Process and Production of Flour Fractions (Fine and Coarse)

The popping process was conducted using hot air, following the methodology of González-Nimi et al. [13]. Briefly, 25 g of grain was popped in an air popper (CPM-150 W, Cuisinart, Glendale, AZ, USA) until the interval between individual grain pops ranged from 3 to 5 s, at which point the popping process was considered complete. The expanded popcorn was then cooled for 5 min before being ground. Then, 25 g of raw grains or popcorn were ground for 1 min with an electric pulverizer mill (SV-MOL-300, Semillas de Vida, Cuernavaca, Morelos, Mexico). After grinding, the flours were sifted through a 425 µm mesh sieve (No. 40, Grupo FIICSA, Ciudad Netzahualcoyotl, Estado de México, Mexico). The flour retained on the sieve was classified as the coarse fraction, and the flour passing through was the fine fraction. These fractions were weighed and stored at 4 °C in sealed polyethylene bags for further analysis. For each hybrid, two independent samples were prepared and tested for further assays.

### 2.3. Estimation of In Vitro Estimated Glycemic Index (eGI) of Popcorn Hybrids

The *in vitro* estimated glycemic index (eGI) was measured using the methodology described by Goñi et al. [14], with some modifications. White bread was used as the reference food for this experiment. Briefly, 400 mg of popcorn flour (fine or coarse fraction) was suspended in 5 mL of phosphate buffer (0.1 M, pH 6.9). The sample was homogenized, and the pH was adjusted to 2.5 with HCl [0.5 M]. Then, 200 µL of pepsin (250 mg/mL, from porcine gastric mucosa, ≥250 U/mg protein, Sigma-Aldrich, St. Louis, MO, USA) was added. The mixture was incubated at 37 °C for 10 min with constant agitation in a water bath. Next, pH was adjusted to 4.7 with sodium acetate buffer (0.4 M, pH 4.7). Subsequently, 300 µL of α-amylase (125 mg/mL, from porcine pancreas, Type VI-B, ≥10 U/mg, Sigma-Aldrich, St. Louis, MO, USA) and 40 µL of amyloglucosidase (3300 U/mL, from the K-DSTRS Digestible and Resistant Starch Assay Kit, Megazyme International, Bray, Ireland) were added, and the mixture was incubated at 37 °C with continuous shaking for 3 h. During incubation, 300 µL of an aliquot was collected at different time points (t = 0, 30, 60, 90, 120, and 180 min) and immediately suspended in cold 80% EtOH (80:20, ethanol:water, *v*/*v*) to deactivate the enzymes. The samples were stored at 4 °C until analysis. All aliquots were centrifuged at 4000 rpm for 5 min at 4 °C, and the supernatants were collected for glucose measurement using the glucose oxidase–peroxidase (GOPOD) assay with a Megazyme kit (K-GLUC, Megazyme International, Bray, Ireland). The assay was performed in duplicate.

The area under the curve (AUC) for the sample reference food and the samples was calculated using GraphPad Prism version 10.6.1 (GraphPad Software, San Diego, CA, USA) (Figure S1). To determine the eGI, the hydrolysis index (HI) was calculated using the following Equation (1):

(1) $$Hydrolysis\;index\;\left(HI\right)=\left(\frac{AUC\;of\;the\;sample}{AUC\;of\;the\;reference\;food}\;\times 100\right)$$

Then the eGI was obtained using Equation (2):

(2) Estimated Glycemic Index (eGI)= 39.71+0.549 HI

### 2.4. Dietary Fiber Analysis

Soluble dietary fiber (SDF), insoluble dietary fiber (IDF), and total dietary fiber (TDF) were determined using a Megazyme kit (K-TDFR-200A, Megazyme International, Bray, Ireland). Briefly, 1 g of popcorn flour (fine or coarse fraction) was incubated with a heat-stable α-amylase solution at 90 °C under continuous stirring for 30 min. Then, the sample was incubated with a protease solution at 60 °C, with continuous stirring for 30 min. After that, the sample was incubated with an amyloglucosidase solution at 60 °C, maintaining continuous stirring for 30 min. The sample was then filtered through a crucible and washed with water at 70 °C. To obtain IDF, the residue collected in the crucible was washed with 95% EtOH and acetone, dried overnight at 70 °C, and weighed. To obtain SDF, the filtrate was precipitated by adding four volumes of preheated 95% EtOH at 60 °C for 1 h. The precipitate was then filtered and washed with 78% EtOH, 95% EtOH, and acetone. The residue was dried overnight at 70 °C and weighed. TDF was calculated as the sum of SDF and IDF. Results were expressed as a percentage. The assay was performed in duplicate.

### 2.5. Phenolic Compounds and Phytosterol Profiles by UPLC-PDA-QDA-ESI+ and UPLC-PDA-ELSD

#### 2.5.1. Quantification of Phenolics

Phenolic compounds were extracted as described by Luzardo-Ocampo et al. [15]. Briefly, extracts of grain and popcorn from both fine and coarse fractions were reconstituted in acetonitrile and analyzed by Ultra-Performance Liquid Chromatography-Photodiode Array-Quadrupole Detector Accuracy-Electrospray Ionization (UPLC-PDA-QDA-ESI+) to quantify ferulic acid and related phenolic compounds. Separation was performed on an Acquity BEH C18 column (2.1 × 100 mm, 1.7 μm) at 50 °C with a flow rate of 0.3 mL/min. The mobile phase consisted of (A) water acidified with 0.1% formic acid (*v*/*v*) and (B) acetonitrile. Gradient elution was performed as follows: 0–0.8 min, 5% B; 0.8–4 min, 5–18% B; 4–8 min, 30% B; 8–12 min, 30–55% B; 12–14 min, 55–78% B; 14–15.5 min, 78–95% B; 15.5–17 min, 95% B; and 17–20 min, 5% B for column re-equilibration. Spectral data were acquired in the 230–550 nm range, and chromatograms were monitored at 320 nm. Commercial authentic standards of *p*-coumaric acid (y = 7545.4x − 14,381, R^2^ = 0.9999) and ferulic acid (y = 8611.4x − 11,523, R^2^ = 0.9999) were used to perform the quantification. *p*-Coumaric acid was quantified against its own curve, whereas ferulic acid, diferulic acid isomers, and the hydroxycinnamic acid derivatives were quantified as ferulic acid equivalents. Calibration curves were prepared for each analytical batch over nine concentration levels from 0.98 to 250 µg mL^−1^.

#### 2.5.2. Quantification of Phytosterols

Phytosterols were extracted as described by Luzardo-Ocampo et al. [15]. Briefly, extracts from grain and popcorn samples, separated into fine and coarse fractions, were reconstituted in acetonitrile, and the content of stigmasterol, campesterol, and β-sitosterol was analyzed by ultra-performance liquid chromatography coupled with a photodiode array detector and an evaporative light scattering detector (UPLC-PDA-ELSD). ELSD parameters were set as follows: 200 gain, 30 psi gas pressure, nebulizer in heating mode at 80% power, and drift tube temperature of 50 °C. Separation was performed on an Acquity BEH C18 column (2.1 × 100 mm, 1.7 μm) at 30 °C with a flow rate of 0.15 mL/min. The mobile phase consisted of (A) methanol and (B) acetonitrile, both acidified with 0.1% formic acid (*v*/*v*). Gradient elution was performed as follows: 0–4 min, 0% B; 4–4.1 min, 0–20% B; 4.1–15 min, 30% B; 15–15.1 min, 30–0% B; 15.1–20 min, 0% B for column re-equilibration. Commercial authentic standards of stigmasterol (y = 10,742x − 113,677, R^2^ = 0.9992), campesterol (y = 727.18x − 279.54, R^2^ = 0.9996), and β-sitosterol (y = 4484.2x − 3447.6, R^2^ = 0.9996) were used to perform the quantification. Calibration curves ranging from 5 to 200 µg/mL were used to quantify stigmasterol and β-sitosterol, while a calibration curve from 5 to 100 µg/mL was used to quantify campesterol.

### 2.6. Antioxidant Capacity Assays

#### 2.6.1. Extraction of Free and Bound Phenolic Compounds

To analyze the antioxidant activity of flour fractions, free and bound phenolic compounds were extracted following the method of Adom & Liu [16], with some modifications. In summary, 250 mg of grain or popcorn flour (fine or coarse fraction) was mixed with 7 mL of methanol (80:20 *v*/*v* methanol:water), incubated under constant agitation, and then centrifuged. For the isolation of free phenolic compounds, the supernatant was recovered, filtered through a 0.45 µm PES membrane (Supor^®^-450, Pall Life Sciences, Ann Arbor, MI, USA), protected from light, and stored at −20 °C until use. To extract bound phenolic compounds from the pellet obtained after free phenolic extraction, alkaline hydrolysis was performed using 2 M NaOH at 90 °C with continuous stirring for 1 h, followed by neutralization with 2 M HCl. Two hexane washes were performed to remove lipids, followed by two ethyl acetate washes to extract the bound phenolic compounds. Both the alkaline hydrolysis and ethyl acetate extraction steps were repeated twice. Ethyl acetate was evaporated to dryness at room temperature. The sample was then resuspended in 80% methanol, filtered through a 0.45 µm PES membrane (Supor^®^-450, Pall Life Sciences, Ann Arbor, MI, USA), protected from light, and stored at −20 °C until use.

#### 2.6.2. ABTS [2,2′-Azino-bis(3-ethylbenzothiazolin)-6-sulfonic Acid] Radical Scavenging Activity

The antioxidant activity was assessed using the ABTS radical method, as described by Re et al. [17]. To generate ABTS+ radicals, a 7 mM ABTS stock solution (Sigma-Aldrich, St Louis, MO, USA) was combined with 2.4 mM K_2_S_2_O_8_ in a 1:1 ratio. This mixture was incubated at room temperature, protected from light, for 12 to 16 h. The resulting ABTS+ radicals were then diluted with absolute methanol to reach an absorbance of 0.700 ± 0.002 at 754 nm. For the case of bound phenolic compounds, a dilution of 1:4 was performed. To assess antioxidant activity, 20 µL of either a grain or popcorn sample was mixed with 180 µL of the ABTS+ dilution and incubated for 6 min. Absorbance was measured at 734 nm using a BioTek Synergy HTX multimode microplate reader (Agilent Technologies, Winooski, VT, USA). The assay was performed in triplicate. The ABTS scavenging activity (%) of grain and popcorn was calculated using the following Equation (3):

(3) $$ABTS\;scavenning\;activity\left(\%\right)=1-\frac{Abs\;of\;sample\;at\;734\;\mathrm{nm}}{Abs\;of\;control\;at\;734\;\mathrm{nm}}\;\times \;100$$

#### 2.6.3. DPPH (2,2-Diphenyl-1-picrylhydrazyl) Radical Scavenging Activity

The antioxidant activity was measured using the free DPPH radical scavenging method [18]. A stock solution of DPPH [100 µM] was prepared with absolute methanol, sonicated for 20 min, and kept away from light. For the case of bound phenolic compounds, a dilution of 1:4 was performed. Then, 20 µL of either a grain or popcorn sample was mixed thoroughly with 180 µL of the DPPH stock solution and incubated in the dark at room temperature for 30 min. Absorbance was then measured at 490 nm with a BioTek Synergy HTX multimode microplate reader (Winooski, VT, USA). The assay was performed in triplicate. The DPPH scavenging activity (%) of grain and popcorn was calculated using the following Equation (4):

(4) $$DPPH\;scavenning\;activity\left(\%\right)=1-\frac{Abs\;of\;sample\;at\;490\;\mathrm{nm}}{Abs\;of\;control\;at\;490\;\mathrm{nm}}\;\times \;100$$

### 2.7. Experimental Design and Statistical Analysis

Two separate samples were tested for each hybrid across all assays. One-way ANOVA was applied to dietary fiber and eGI. For phenolic compounds, phytosterols, and antioxidant activity, a two-way ANOVA was performed to evaluate the effects of hybrid, fraction, and their interaction. Mean comparisons were conducted using Tukey’s test to determine significant differences among samples at the *p* < 0.05 significance level. These analyses were performed using JMP software (version 19.0.5, SAS, Cary, NC, USA).

For the multivariate analyses of the phenolic profile, the free and bound phenolic profiles were concatenated into a single matrix and autoscaled to a zero mean and unit variance. Principal component analysis (PCA) was used as an exploratory approach to visualize the overall relationships among samples. Partial least squares discriminant analysis (PLS-DA) and orthogonal PLS-DA (OPLS-DA) were subsequently applied to discriminate between the control and hybrids. Model performance was assessed by leave-one-out cross-validation and permutation testing (1000 permutations) and discriminant compounds were identified by a variable-importance-in-projection (VIP) score greater than 1. These multivariate analyses were performed in Python 3 using NumPy, SciPy, pandas, and scikit-learn libraries.

The correlation between the eGI of popcorn samples and their compositional variables (including IDF, SDF, total free phenolics, total bound phenolics, total phytosterols, and antioxidant capacity) was assessed using both Pearson and Spearman correlation coefficients. Each sample’s average was treated as a single observation, leading to a total of six observations (*n* = 6; five hybrids and one commercial popcorn sample). Pearson’s was used to evaluate linear relationships, while Spearman’s served as a non-parametric check of the robustness of the correlation. To explore multivariate relationships among these variables, PCA was performed using autoscaled variables (mean-centered and standardized to unit variance). The eGI was added as a supplementary, illustrative variable that projects onto the PCA space without influencing the principal components. This facilitated visualization of the relationship between eGI and the overall compositional profile of the popcorn hybrids. These analyses were performed using JMP software (version 19.0.5, SAS, Cary, NC, USA).

## 3. Results and Discussion

### 3.1. Estimation of the In Vitro Estimated Glycemic Index (eGI)

The *in vitro* eGI values for the popcorn samples are presented in Table 1. Popcorn C had the highest eGI, while Popcorns 2, 4, and 5 had the lowest. According to the GI scale, all the hybrids have a low eGI (<55), which can be considered low-GI foods, while Popcorn C had a medium eGI (56–69). Comparable to previous reports on other maize snacks, particularly extrudates of blue maize, which showed an eGI of 53.06 and were more closely related to the anthocyanin and phenolic compound profiles than to fiber content [8], no differences were observed.

The glycemic index of foods can be influenced by the structural organization of the food matrix and its chemical composition. Several intrinsic factors, such as dietary fiber, resistant starch, phenolic compounds, lipids, and proteins, can reduce starch digestibility through various mechanisms, including limiting enzyme accessibility, interacting with starch, or inhibiting digestive enzymes. Additionally, extrinsic factors such as particle size, cooking methods, and food processing influence starch hydrolysis and, consequently, the GI [5]. Previous studies have shown that cooking with gradual dry heat can preserve crystalline starch regions, which promote the formation of starch complexes with other molecules, thereby lowering GI [19,20].

In this study, variations in eGI among popcorn hybrids likely reflect differences in their intrinsic composition and structural arrangement. The lower eGI found in Popcorns 2, 4, and 5 may result from multiple factors that limit starch hydrolysis, such as differences in dietary fiber, phenolic compounds, and starch matrix organization. Conversely, the higher eGI in Popcorn C may indicate greater accessibility of starch to digestive enzymes or a lower contribution of components that slow starch breakdown. These factors probably act together rather than separately, and their impact may vary across hybrids. Since all popcorn hybrids in this study were subjected to the same popping conditions, the differences in eGI are more likely to reflect hybrid-specific compositional and structural characteristics than differences in the cooking process. Therefore, the compositional characteristics of the hybrids were further analyzed to identify which factors might have contributed to the variations in eGI.

### 3.2. Dietary Fiber Content

**Figure 1:**
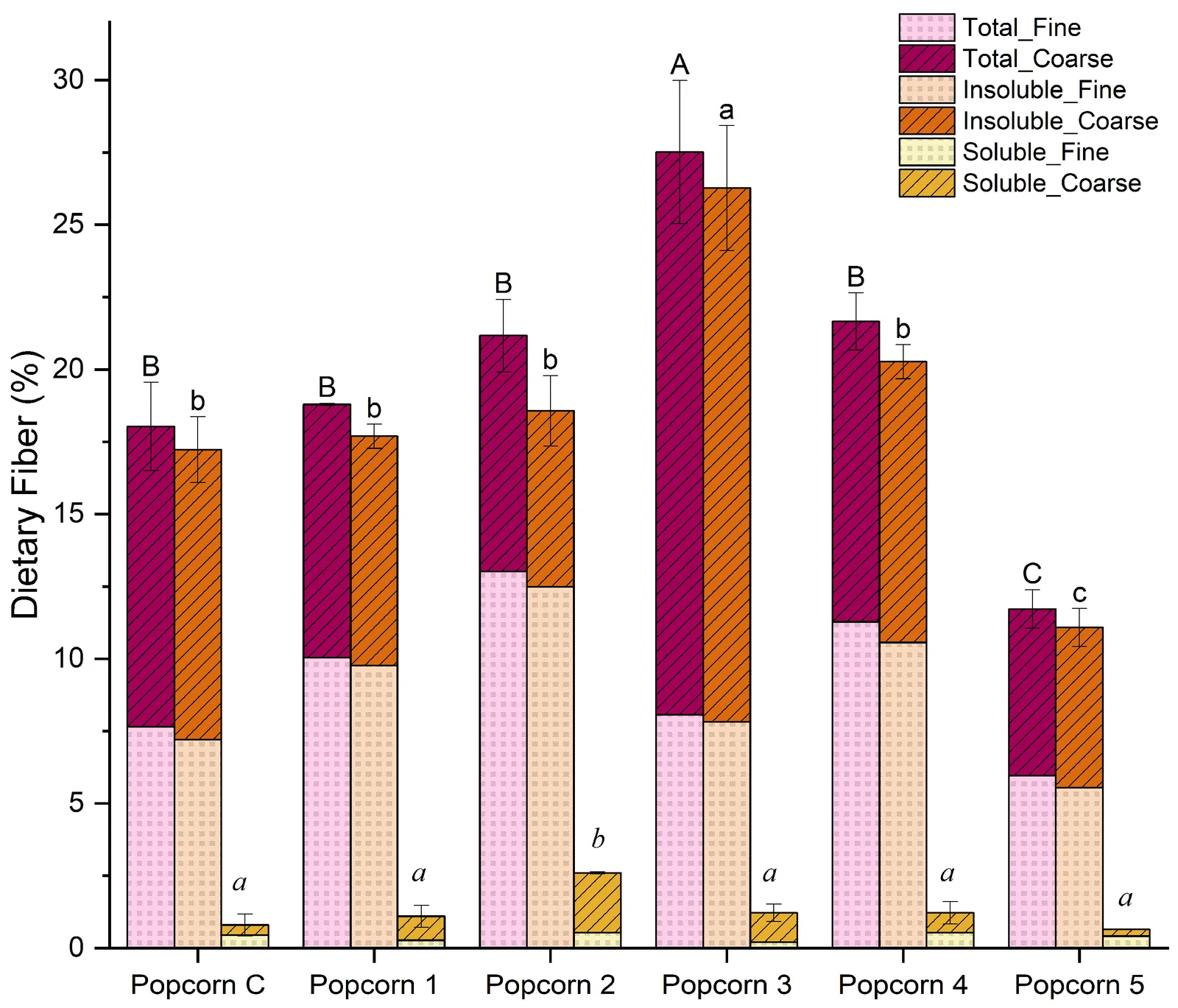
Dietary fiber content of whole popcorn. Results are expressed as means of duplicates ± SD (Standard deviation). Different uppercase letters indicate significant differences in Total Dietary Fiber (TDF), lowercase letters indicate significant differences in Insoluble Dietary Fiber (IDF), and italicized letters indicate significant differences in Soluble Dietary Fiber (SDF) content among samples (*p* < 0.05).

Popcorn is recognized as one of the snacks with the highest dietary fiber content, with approximately 15.1% TDF reported for air-popped popcorn in the literature [21]. In the present study, TDF content in the whole popcorn matrix ranged from 11.7% to 27.4%, with the highest values approaching twice those previously reported. Among the samples evaluated, Popcorn 3 had the highest TDF (27.4%) and IDF contents (Figure 1), whereas Popcorn 5 showed the lowest dietary fiber content. In general, the coarse fraction contributed the largest proportion of TDF, particularly in Popcorn 3, reflecting greater retention of the pericarp in this fraction, the main source of dietary fiber in the kernel. Regarding SDF, Popcorn 2 presented the highest content (2.6%) among the other samples, while Popcorn 3 showed no significant differences compared with the other hybrids.

These results are consistent with previous studies on maize-based snacks. Çetin-Babaoğlu et al. [8] analyzed the dietary fiber content of pigmented maize snacks of blue, red, and yellow maize, obtaining TDF values of 26.8 g/100 g for the blue maize and 23.3 g/100 g for the yellow maize snacks, with blue maize presenting the highest TDF and IDF content. Consistent with our findings, they observed no significant differences in SDF among the pigmented maize snacks, suggesting that maize pigmentation had little influence on the distribution of soluble and insoluble dietary fiber. TDF values obtained in the present study are therefore comparable to those reported for maize snacks, although the contributions of the fiber fractions varied among popcorn hybrids.

Although Popcorn 3 had the highest TDF and IDF contents, it was not the sample with the lowest eGI. Instead, Popcorn 2, which had the highest SDF content, exhibited the lowest eGI. In contrast, although Popcorn 5 had the lowest dietary fiber content, it also presented a low eGI. These findings suggest that the glycemic response is influenced not only by TDF but also by its physicochemical characteristics and composition of dietary fiber. SDF increases the viscosity of digested starch, forming a gel matrix that slows glucose diffusion and absorption while restricting the access of α-glucosidase and α-amylase to starch substrates [22]. In contrast, IDF acts mainly as a physical barrier, limiting starch gelatinization, water uptake, and granule swelling, and can further inhibit α-amylase and α-glucosidase through enzyme adsorption or the presence of fiber-bound phenolic compounds [22,23]. Consequently, differences in the relative proportion of SDF and IDF among the popcorn hybrids may partially explain their distinct responses. The lower eGI observed in Popcorn 2 is consistent with its higher SDF content, whereas the absence of a similar response in Popcorn 3 suggests that other factors, such as popcorn matrix compounds and their interactions, may play a more important role in modulating starch digestibility.

### 3.3. Phenolic Profile by UPLC-PDA-QDA-ESI+

#### 3.3.1. Free Phenolic Compounds

In general, six free phenolic compounds were identified in grain, comprising two monomeric phenolic acids and four hydroxycinnamic acid amides (HCAAs) (Table S1). The monomeric phenolic acids included p-coumaric acid (p-CA) and ferulic acid (FA), consistent with previous research that described these compounds as the main monomeric phenolic acids in the free fraction of yellow maize [24]. The HCAAs consisted of feruloylputrescine (FP), diferuloylputrescine (DFP), dicoumaroylputrescine (DCP), and N-feruloyl-N’-coumaroylputrescine (FCP). To our knowledge, this is the first report identifying these HCAAs in this sample type.

p-CA was the dominant free monomeric phenolic acid, ranging from 29.3 to 74 µg p-coumaric acid/g grain (Table S2), with the highest concentration observed in Grain C. While FA showed concentrations between 5.6 and 10.3 µg ferulic acid/g, there was no significant difference among samples. Both monomeric phenolic acids were consistently higher in the fine fraction, whereas FP, DFP, DCP, and FCP predominated in the coarse fractions. DFP was the major HCAA, ranging from 14.4 to 99 µg ferulic acid equivalents/g, with the highest value detected in the coarse fraction of Grain C.

The contrasting distributions of monomeric phenolic acids and HCAAs across particle size fractions likely indicate their specific locations within kernel tissues. The higher abundance of monomeric phenolic acids in the fine fraction suggests that particle-size reduction facilitates their extraction by increasing the exposed surface area, whereas HCAAs tend to remain associated with the recalcitrant cell wall matrix of the pericarp. Similar behavior has been reported for the mechanical processing of corn bran, demonstrating that intact bran serves as a reservoir for phenolic compounds, while micrometric pulverization forces their mechanical release into the soluble fraction [25,26].

Beyond its distribution within the grain, the predominance of p-CA may also have nutritional implications, as hydroxycinnamic acids have been associated with reduced starch digestibility and a lower postprandial glycemic response. These effects have been attributed to their ability to interact with starch and digestive enzymes such as α-amylase and α-glucosidase, thereby delaying carbohydrate breakdown. Li et al. [27] reported that coumaric acid isomers, especially p-CA, form inclusion complexes with starch helices more effectively, thereby increasing resistant starch (RS) levels and lowering the eGI. Although free phenolic acids comprised only a small portion of the total phenolic compounds in this study, differences in their concentrations among hybrids, along with other grain components, could have contributed to the differences observed in eGI.

In the case of popcorn, nine free phenolic compounds were identified, including three monomeric phenolic acids (cis-p-coumaric acid (c-CA), trans-p-coumaric acid (t-CA), and FA) and six HCAAs (FP, four geometric isomers of diferuloylputrescine [DFP-1, DFP-2, DFP-3, and DFP-4], and FCP) (Table S1).

Similar to grain, free monomeric phenolic acids were consistently higher in the fine fraction. The highest c-CA concentration was 4.7 ± 0.2 µg p-CA/g popcorn, found in the fine fraction of Popcorn 5 (Table S3). Meanwhile, the highest t-CA concentration (5.7 ± 0.1 µg p-CA/g) was observed in the fine fraction of Popcorn C. FA had its highest value in the fine fraction of Popcorn 4. Likewise, p-coumaric acid (combined c-CA + t-CA) was the predominant monomeric acid. In the case of HCAA, the major concentration of compounds was in the coarse fraction, although its composition differed from that of grain because four DFP isomers and one co-eluting FCP+DFP-3 fraction were detected after popping.

Comparison between grain and popcorn showed a significant decrease in free monomeric phenolic acids after popping. For instance, the total free p-CA in Grain C decreased significantly (*p* < 0.05) from 73.3 to 8.5 µg/g after popping (88.4%). Conversely, changes in HCAAs varied across hybrids because not all compounds appeared in every fraction, indicating that individual putrescine conjugates responded differently to the popping process. These distinct responses likely reflect the combined effects of thermal degradation, isomerization, and differential release from the kernel matrix during popping. Although the free phenolic fraction was modified by thermal processing, the bound phenolic fraction represents the major reservoir of hydroxycinnamic acids in cereals and is therefore expected to play a more prominent role in modulating starch digestibility and, consequently, the differences in eGI observed among hybrids.

#### 3.3.2. Bound Phenolic Compounds Fraction Identification and Quantification

Nine bound phenolic compounds were identified in grain, including two monomeric phenolic acids (CA and FA), two HCAAs (FCP and DFP), and five diferulic acid (DFA) isomers (Table S1). In contrast with the free fraction, the bound fraction was dominated by DFA isomers, which are formed through oxidative coupling of ferulate monomers within the cell wall [28].

Bound phenolic compounds were more abundant than free phenolic compounds, consistent with the extensive esterification of phenolic acids to the cell wall matrix [29,30]. Particularly in grains, FA was the most predominant bound monomeric phenolic acid, with the highest concentration of 3074.7 µg ferulic acid equivalents/g obtained in the coarse fraction of Grain 2 (Table S4), whereas p-CA reached a highest concentration of 237.3 ± 22.8 µg CA/g in the same fraction of Grain 2. DFA isomers were also concentrated in the coarse fraction, with 5,5′-DFA and 8-O-4′-DFA as the predominant isomers, both reaching their highest concentrations in the coarse fraction of Grain 2. Total DFA ranged from 379.5 to 698.8 µg ferulic acid equivalents/g, with the highest concentration observed again in the coarse fraction of Grain 2. The predominance of bound phenolics in the coarse fraction is consistent with the contribution of the pericarp to this fraction, where ferulate residues are esterified to arabinoxylans and can undergo oxidative coupling to form DFA cross-links that reinforce the cell-wall network [28,31]. This structural organization may affect starch digestibility by increasing the barrier to digestive enzymes’ access to starch granules.

Beyond its structural function in the cell wall, bound FA may also influence starch digestibility. Phenolic acids can interact with starch and digestive enzymes, potentially changing starch structure and enzyme access during digestion. [7]. In particular, recent studies show that interactions between FA and starch can decrease starch hydrolysis, although the magnitude of this effect varies depending on the starch matrix and the nature of the phenolic–starch interaction [32,33]. Therefore, the high abundance of bound FA observed across the popcorn hybrids, along with differences in DFA accumulation and cell-wall organization, may have contributed to variations in starch accessibility and estimated glycemic index (eGI) among samples. However, because the present study did not directly evaluate FA–starch interactions or enzyme inhibition, these relationships should be viewed as potential contributing mechanisms rather than direct causal effects.

The bound phenolic profile remained consistent after popping, although the amounts and distribution of individual compounds changed significantly. These differences should be interpreted considering the moisture loss during popping, which can lead to an apparent increase in phenolic compound concentration. Popcorn’s bound phenolic profile included two monomeric phenolic acids (p-CA and FA), four HCAAs (FP, FCP, DCP, and DFP), and five DFA isomers (8,5′-DFA OPC, 5,5′-DFA, 8-O-4′-DFA, 8,5′-DFA BZF, and 8,8′-DFA). Bound FA remained the predominant phenolic acid after popping, reaching up to 6764.9 µg/g popcorn, whereas bound p-CA reached a maximum of 469.9 µg/g popcorn (Table S5). DFA isomers also increased markedly after popping, with 5,5′-DFA and 8-O-4′-DFA remaining the major dimers. Among the experimental hybrids, total DFA in the fine fraction ranged from 814.7 to 1304.7 µg/g popcorn.

In contrast to raw grain, where bound phenolics were preferentially concentrated in the coarse fraction, popping caused a marked redistribution toward the fine fraction across all genotypes. For example, bound FA in the fine fraction of Popcorn C increased 5.7-fold relative to Grain C, whereas total DFA increased 6.6-fold. This inversion likely reflects the disruption of the pericarp cell wall during kernel expansion, which redistributes wall-associated phenolics into smaller particle-size fractions, as previously reported for thermo-mechanically processed cereals [31,34]. The concentration values are consistent with concentrations reported for the tissue in which these compounds are compartmentalized. Ester-linked ferulic acid (FA) in the isolated pericarp cell wall of popcorn inbred lines ranges from 13,710 to 18,070 µg/g [35], whereas bound *p*-coumaric acid (CA) and ferulic acid (FA) derivatives in the whole grain of oily ‘Everta’ hybrids range from 1621.75 to 1970.94 µg/g [15].

#### 3.3.3. Multivariate Integration of the Hybrid, Flour Fraction, and Processing Effects on the Phenolic Profile

To obtain an integrated overview of phenolic composition, free and bound fractions of each sample were combined and analyzed by PCA (Figure S2). The PCA showed a consistent separation between the control and the hybrids while preserving the differentiation between raw grain and popped samples, indicating that the major compositional patterns were maintained after processing.

**Figure 2:**
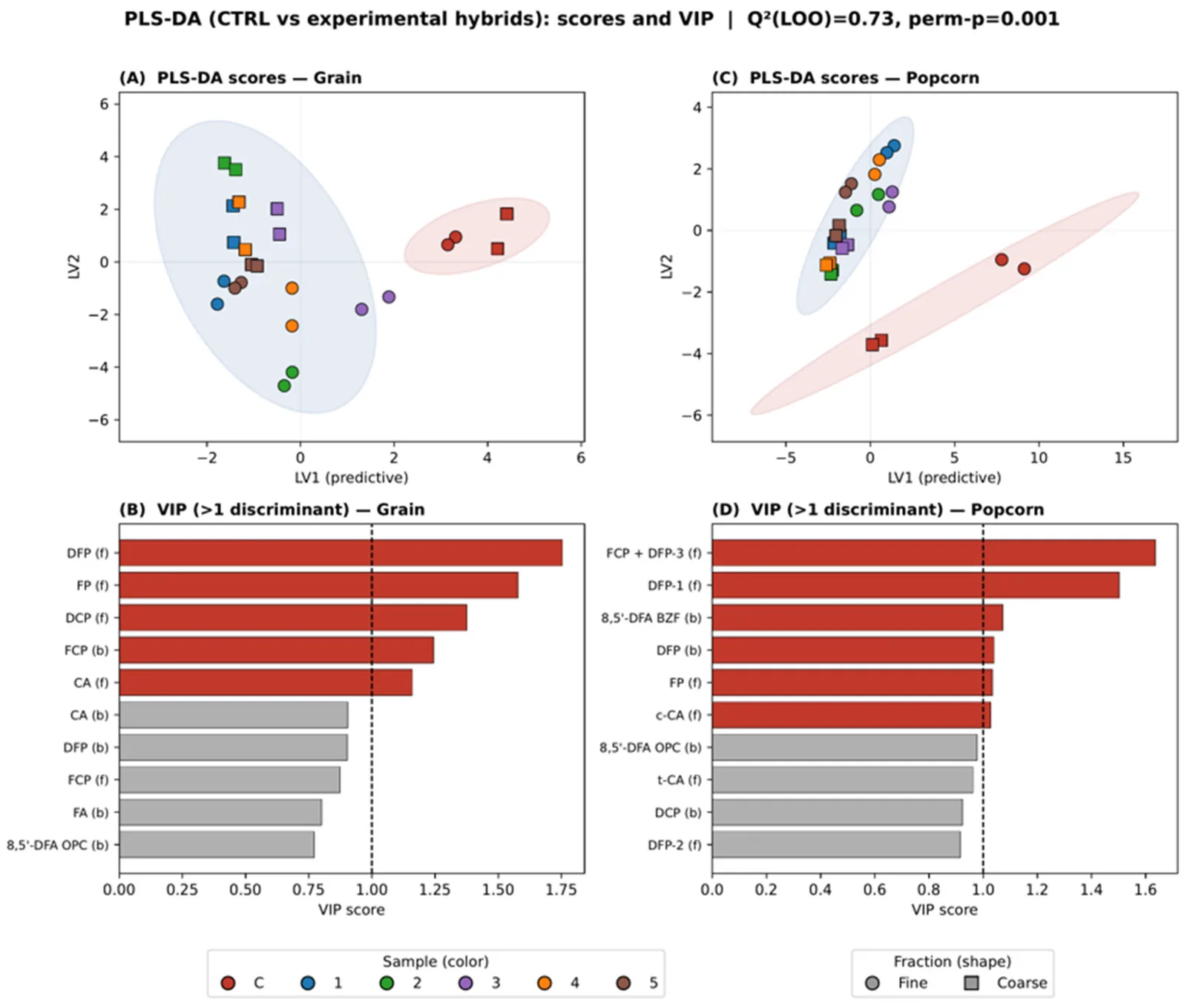
PLS-DA discriminating the control (CTRL) from the hybrids (M1–M5). (**A**,**C**) score plots for grain and popcorn (ellipses: 95% confidence regions of each class); (**B**,**D**) corresponding variable-importance-in-projection (VIP) scores, with compounds above the VIP = 1 threshold (dashed line) highlighted. Suffixes (f) and (b) denote compounds quantified in the free and in the cell-wall-bound fraction, respectively. Models were validated by leave-one-out cross-validation (Q^2^ = 0.73) and label-permutation testing (*p* = 0.001).

To further evaluate the discrimination between the control and the hybrids, a supervised PLS-DA (Figure 2) and an OPLS-DA were constructed. The model showed strong predictive performance for both raw grain and popped popcorn (Q^2^ = 0.73), with balanced classification accuracies ranging from 0.88 to 0.97 and significant permutation test results (*p* = 0.001), indicating that the observed separation was unlikely to arise by chance.

The compounds contributing most to this separation (VIP > 1) included the free putrescine conjugates DFP, FP, and DCP, together with bound FCP in grain, whereas the popcorn samples were primarily differentiated by free DFP isomers and the bound 8,5-DFA benzofuran isomer. These compounds were therefore identified as the principal markers distinguishing the control from the hybrids.

The predominance of hydroxycinnamic acid amides among the discriminant variables supports the hypothesis that the experimental hybrids retain a broader spectrum of defense-related phenolics than the commercial control, consistent with previous reports describing a reduction in these metabolites during maize domestication [36,37]. The persistence of these compounds after popping further suggests that they are stable compositional markers with potential relevance to the development of functional popcorn-based foods.

### 3.4. Phytosterols Profile by UPLC-PDA-ELSD

In the grain samples, total phytosterol content was significantly higher in the fine fractions than in the corresponding coarse fraction for almost all the hybrids, except for Grain 4, which showed no significant difference between fractions (Table S6).

In the fine fraction of grain samples, Grain C exhibits the highest concentration of total phytosterol content (118.6 μg/g sample), while the total content of grain hybrids ranges between 63.2 and 87.8 μg/g sample. In the coarse fraction, Grain 3, Grain 4, and Grain 5 had the highest total phytosterol content, with concentrations ranging from 50.8 to 54.5 μg/g sample. These differences suggest that phytosterols are not uniformly distributed across grain fractions, which may be associated with the heterogeneous distribution of lipids among the anatomical tissues of the grain. Variation among hybrids was also observed, suggesting that genotype may influence phytosterol accumulation and distribution.

After popping, total phytosterol concentrations were significantly higher than those measured in the corresponding grain samples, in most cases. In general, the fine fractions have higher phytosterol content than the coarse fraction. All popcorn samples showed higher phytosterol content than the control popcorn (Popcorn C). The highest total phytosterol content was observed in the fine fraction of Popcorn 5 (198.2 ± 21.8 µg/g), with β-sitosterol (94.3 ± 1.7 µg/g) as the most abundant phytosterol, followed by campesterol (83.4 ± 20.4 µg/g) and stigmasterol (20.5 ± 0.2 µg/g). β-Sitosterol, campesterol, and stigmasterol are among the major phytosterols commonly found in cereal grains and plant-derived foods and are recognized as bioactive components with potential health-promoting effects [38]. Differences in the relative abundance of individual phytosterols were also observed among popcorn hybrids. For example, in Popcorn C, β-sitosterol was about 7.3- and 4.2-times higher than stigmasterol and campesterol, respectively, while this distribution varied among the experimental hybrids, indicating genotype-dependent differences in phytosterol composition.

The higher phytosterol concentrations observed in popcorn than in grain may partly reflect moisture loss during popping, which can concentrate non-water components in the popping product. Therefore, these findings should be interpreted as differences in phytosterol concentration rather than as direct evidence that popping increased the total amount of phytosterols. However, the variation among different hybrids and fractions suggests that both genetic factors and processing methods can affect the phytosterol profile in popcorn. In addition to their role in plant membrane structure, phytosterols are linked to cholesterol metabolism, antioxidant and anti-inflammatory responses, and metabolic regulation [38,39]. Although phytosterols are not directly involved in starch hydrolysis, their possible effects on metabolic regulation could indirectly influence the overall physiological response to popcorn consumption. Therefore, phytosterols, along with dietary fiber, phenolic compounds, and other components of the grain matrix, may contribute to the distinct nutritional and functional characteristics of the popcorn hybrids.

### 3.5. Antioxidant Activity Assays

**Figure 3:**
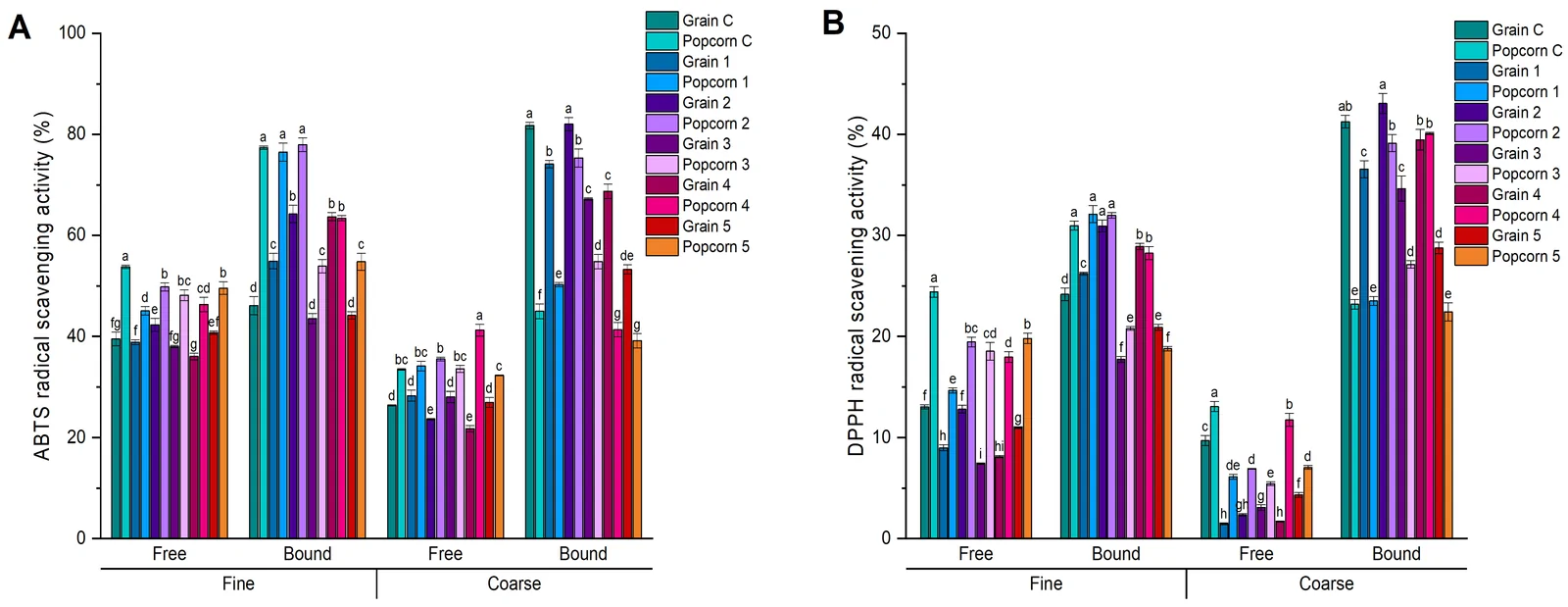
Percentage of antioxidant activity of the fine and coarse fractions of grain and popcorn samples using the (**A**) ABTS radical scavenging and (**B**) DPPH radical scavenging tests. The results are expressed as the means of duplicates ± SD (standard deviation). Different letters within each fraction and phenolic compound column segment indicate significant differences (*p* < 0.05) by the Tukey test.

The antioxidant activity of the free and bound phenolic compounds of fine and coarse fractions of grain and popcorn samples is represented in Figure 3. Regarding ABTS antioxidant activity (Figure 3A), it was higher in bound phenolic compounds than in free phenolic compounds in both grain and popcorn samples. This trend is consistent with the phenolic profile, which showed that bound phenolics represented the major phenolic fraction, particularly FA and DFA derivatives. In grain, these compounds were predominantly concentrated in the coarse fraction, reflecting their association with the pericarp cell wall matrix. However, after popping, bound phenolics showed a marked redistribution toward the fine fraction.

For ABTS activity (Figure 3A), free phenolic compounds in the fine fraction of grain showed antioxidant activity values ranging from 37.9% to 42.2%, whereas Popcorn C exhibited the highest activity among the popcorn samples (53.7%). The higher activity observed in the fine fraction may be partly related to the predominance of free monomeric phenolic acids in this fraction, particularly p-CA. In contrast, HCAAs were mainly detected in the coarse fraction of grain. For bound phenolics, most grain samples showed similar antioxidant activity, although Grain 2 differed from the other hybrids. This sample also exhibited the highest concentration of bound FA, together with high concentrations of DFA isomers, suggesting that the abundance of these compounds may have contributed to its antioxidant response. However, the activity should not be attributed to FA alone because several phenolic acids, HCAAs, and DFA isomers were simultaneously present.

In popcorn, Popcorns C, 1, and 2 exhibited the highest ABT activity in the bound fraction, reaching about 77.9%. This is notable because popping significantly altered the phenolic profile. Although free monomeric phenolic acids decreased post-popping, bound FA and DFA increased considerably and shifted toward the fine fraction. For instance, bound FA in the fine fraction of Popcorn C was 5.7 times higher than in Grain C, and total DFA increased 6.6 times. These changes could explain why several popcorn samples retained high antioxidant activity despite a decline in free phenolic acids.

Differences between grain and popcorn were also evident in the coarse fraction. Grain 4 showed the lowest ABTS activity for free phenolic compounds, whereas its corresponding popcorn sample, Popcorn 4, exhibited the highest activity for this fraction (41.2%). For bound phenolics, most grain samples showed higher activity than their popcorn counterparts, except for Popcorn 2, which showed 39.1% inhibition, close to the 43.0% observed for Grain 2. These contrasting responses indicate that the effect of popping on antioxidant activity was genotype- and fraction-dependent. Such variability is consistent with other study that reports differences in antioxidant responses among popcorn hybrids after thermal processing [3].

The DPPH assay (Figure 3B) showed patterns broadly comparable to those observed with ABTS, although antioxidant activity values were generally lower. For free phenolic compounds in the fine fraction, grain samples showed greater variability, whereas popcorn samples showed relatively similar antioxidant activity among hybrids, with Popcorn C showing the highest activity. For the bound fraction, Grain 2 and Popcorn 2 showed similar DPPH activity. In the coarse fraction, free phenolic compounds generally showed higher antioxidant activity in popcorn than in the corresponding grain samples. Conversely, bound phenolics generally exhibited higher activity in grain than in popcorn, except for Popcorn 4, which showed a response similar to that of Grain 4. The genotype-dependent behavior observed here agrees with the findings of Vukadinovic et al. [3], who reported that DPPH antioxidant activity changed differently among popcorn hybrids after microwave popping, with only some hybrids showing an increase after expansion.

The lower antioxidant activity values observed with DPPH compared with ABTS can be attributed to differences in the chemical mechanism and reaction environments of the two assays. ABTS can react with both hydrophilic and lipophilic antioxidants, whereas DPPH responds better to hydrophobic antioxidants, so the mechanisms of action of these two antioxidants can differ depending on the compounds analyzed [40]. Consequently, differences between ABTS and DPPH responses may reflect the heterogeneous composition of the phenolic fractions. Previous studies in maize have also demonstrated that the sensitivity of antioxidant assays depends on the phenolic composition and genotype. Kim et al. [41] reported that DPPH and FRAP were more closely associated with anthocyanin content, whereas ABTS showed a stronger relationship with total phenolic content in purple waxy maize. Similarly, Ramírez-García et al. [42] demonstrated that the response of antioxidant assays varied according to maize grain color and composition.

The phenolic profile provides additional support for interpreting these antioxidant responses. Hydroxycinnamic acids such as FA and p-CA can contribute to radical-scavenging activity through their hydroxyl groups, although their antioxidant contribution depends on their concentration, chemical form, and surrounding matrix [40]. In this study, FA was the most common bound monomeric phenolic acid in the grain, reaching its highest concentration in the coarse fraction of Grain 2. DFA isomers were also notably abundant in this fraction. After popping, bound FA remained the dominant phenolic acid, while DFA isomers increased significantly, shifting toward the fine fraction. This suggests that the antioxidant activity in the bound fraction is likely due to a combination of several hydroxycinnamic acid derivatives rather than a single compound. This interpretation is consistent with previous reports associating higher phenolic content with increased antioxidant capacity in maize and popcorn [4,24].

The differences observed between grain and popcorn may also be related to structural changes induced by popping. Previous studies have reported that thermal processing can modify the accessibility of phenolic compounds by disrupting the cell-wall structure and promoting the release or redistribution of compounds associated with the kernel matrix [31,34]. In the present study, this effect was particularly evident for the bound fraction, because phenolics that were predominantly associated with the coarse fraction in raw grain became more abundant in the fine fraction after popping.

The antioxidant results show that antioxidant capacity is affected by the combined effects of genotype, phenolic composition, chemical form, particle-size fraction, and popping. The reduction in free monomeric phenolic acids after popping did not necessarily result in a decrease in antioxidant activity, likely due to the simultaneous increase and redistribution of bound phenolic compounds, especially FA and DFA. Therefore, the antioxidant potential of popcorn appears to depend not only on total phenolic content but also on its chemical form and its association with the kernel matrix.

### 3.6. Correlation Analysis and Principal Component Analysis (PCA)

**Figure 4:**
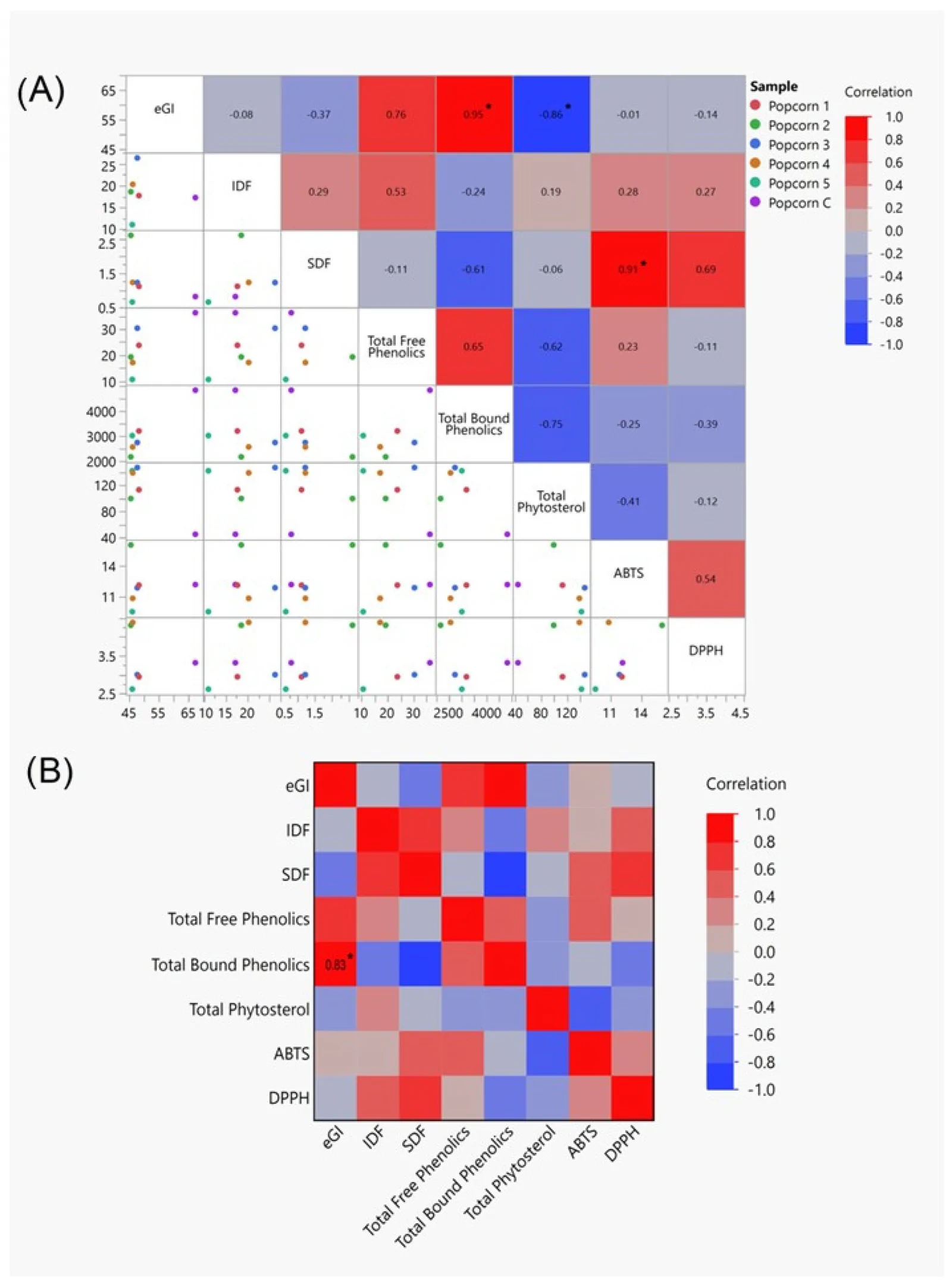
Correlation analysis between eGI and the phytochemical composition of popcorn samples (*n* = 6; Five hybrids and a commercial popcorn sample). (**A**) Pearson correlation analysis with scatter plots illustrating relationships between variables, and correlation coefficients shown by the color scale. (**B**) Spearman rank correlation heat map. Red and blue indicate positive and negative correlations, respectively, with color intensity reflecting the strength of the correlation. Asterisks denote statistically significant correlations (*p* < 0.05).

**Figure 5:**
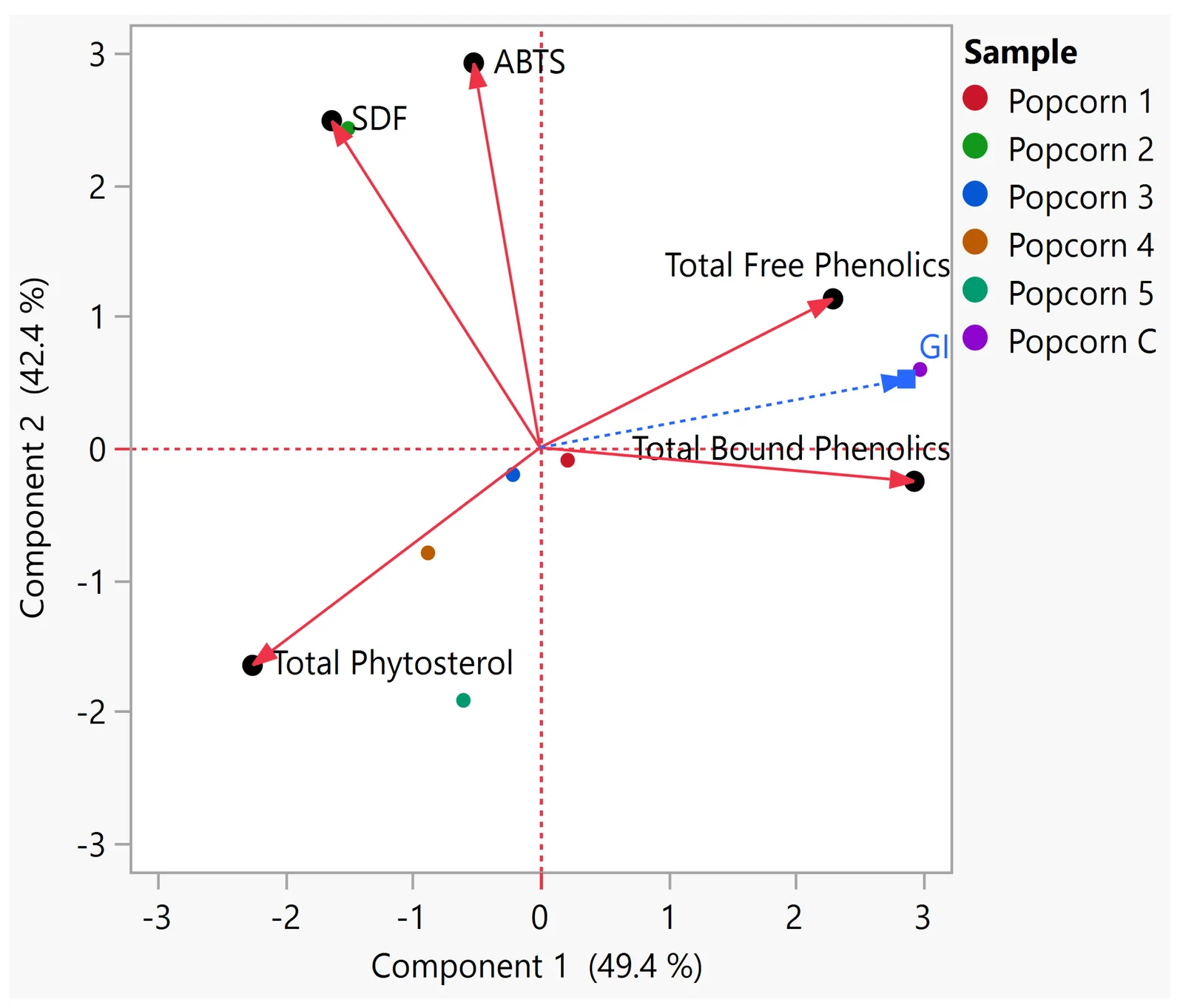
Biplot of the principal component analysis (PCA). The first two components explained 49.4% and 42.4% of the variance, respectively. Arrows indicate the strength of the variables, with their lengths indicating how much each trait contributes to the two PCA components.

The correlation and multivariate analyses revealed relations between the phytochemical composition of the popcorn samples (*n* = 6: five hybrids and one commercial popcorn sample) and their eGI (Table S7). Among the variables evaluated, total bound phenolics showed the strongest correlation with eGI (Figure 4), whereas SDF showed an inverse but non-significant correlation. Total phytosterols tended to be inversely related to eGI, with a significant Pearson correlation; however, this tendency was not supported by Spearman’s analysis. In contrast, ABTS and DPPH showed only a slight correlation with eGI. The PCA (Figure 5) provided a complementary view of these relationships, showing that eGI aligned similarly with total free and total bound phenolic compounds, whereas it was opposite to SDF and total phytosterols. Our results suggest that differences in eGI were associated with the integrated phytochemical composition of the popcorn matrix rather than with a single bioactive compound.

Total bound phenolic compounds exhibited the strongest correlation with eGI, with significant relationships observed in both Pearson (r = 0.9476, *p* = 0.0040) and Spearman (ρ = 0.8286, *p* = 0.0416) analyses. The consistent results across these correlation methods, together with the PCA, indicate an association between bound phenolics and eGI in the samples analyzed. However, this association should be interpreted with caution because of the small sample size (*n* = 6). Moreover, this positive correlation does not imply that bound phenolics directly increase the GI; instead, it may indicate differences in the structural organization of the popcorn grain matrix. FA and other phenolics are linked to cereal cell wall components such as arabinoxylans, while diferulate formation contributes to cross-linking within the cell wall [43]. Variations in bound phenolic levels may partly reflect differences in cell wall composition and organization, which could affect starch accessibility. Further studies incorporating microscopic and structural analysis of the popcorn grain matrix are necessary to assess if variations in cell wall organization and phenolic cross-linking influence starch accessibility and, as a result, the differences observed in eGI.

This interpretation is consistent with our previous research on yellow popcorn hybrids, where variations in their phytochemical profiles were linked to starch accessibility and digestion [13]. That study suggested that starch-phenolic interactions could contribute to these differences. Notably, the phenolic profile was found to be more influential than the total phenolic content. The current results build on these findings by showing that total bound phenolics strongly correlate with eGI, especially when considering phenolic compounds, dietary fiber, phytosterols, and antioxidant variables together. Therefore, the positive correlation observed here does not imply that individual phenolics directly enhance starch digestion or GI. Instead, the level of bound phenolics likely reflects the overall phenolic and structural traits of the grain. This aligns with evidence indicating that the impact of phenolic compounds on starch digestibility depends on their chemical identity, concentration, and how they interact with starch and the surrounding matrix [32,33].

SDF showed a consistent inverse relationship with eGI in both Pearson (r = −0.3710) and Spearman (ρ = −0.4282) analyses, although neither was statistically significant. Notably, Popcorn 2 had the lowest eGI and the highest SDF content. In PCA, SDF was oriented in the opposite direction of eGI, aligning with the hypothesis that SDF reduces starch accessibility and slows digestibility. Recent studies in corn starch systems suggest that SDF can modify starch structure, decreasing its digestibility and eGI [44]. Therefore, SDF might partly explain the lower eGI in Popcorn 2, although current data do not confirm SDF as an independent predictor of eGI.

The combined Pearson, Spearman, and PCA results suggest that eGI is linked to a complex nutritional phenotype encompassing phenolic compounds, dietary fiber, and potentially the grain’s structural organization. The strong correlation between bound phenolics and eGI, along with the inverse trend observed with SDF, suggests that the interaction between phytochemicals and the physical matrix surrounding starch may be more significant than the total phenolic or antioxidant content. These observations complement previous research and support the idea that variations in popcorn GI are related to the grain’s chemical composition and the organization of its matrix. Nevertheless, since the current correlations were based on specific samples (five hybrids and a commercial popcorn sample; *n* = 6), these findings should be considered as preliminary. The distinct profile of the commercial popcorn sample may have influenced the strength of some correlations. Consequently, both correlation analysis and PCA should be regarded as exploratory. More studies involving a broader range of hybrids are necessary to clarify the specific roles of individual phenolic compounds and fiber fractions in influencing glycemic response.

## 4. Conclusions

This study demonstrates that the food matrix and phytochemical composition of popcorn hybrids are important factors to consider when evaluating differences in eGI. The five yellow popcorn hybrids had lower eGI values than the commercial popcorn control, which showed a medium eGI. There were notable differences in the distribution of dietary fiber, phenolic compounds, phytosterols, and antioxidant activity between the fine and coarse fractions. These findings emphasize that popcorn’s nutritional properties depend not only on the levels of individual compounds but also on how these compounds are distributed within the grain matrix.

Among the components analyzed in the matrix, SDF was identified as a relevant factor that may contribute to differences in the glycemic response. Popcorn 2 showed the highest SDF levels and the lowest eGI, supporting the idea that SDF may limit starch accessibility and hydrolysis. However, the lack of a significant association between SDF and eGI indicates that the glycemic response is likely influenced by the combined effects and interactions of multiple components within the popcorn matrix. The phenolic profile showed a variety of free and bound compounds, such as hydroxycinnamic acids, diferulic acids, and hydroxycinnamoylputrescines. The differences in the distribution of these compounds, along with the patterns observed in ABTS and DPPH assays, suggest that the functional properties of the entire matrix might influence the glycemic response more than total phenolic content alone. Likewise, hybrids with higher phytosterol content generally exhibited lower eGI values compared to the commercial control. This suggests that phytosterol content might be an important trait to consider for future selection of popcorn hybrids with improved nutritional qualities.

Although Pearson and Spearman correlation analyses did not identify statistically significant individual associations between eGI and most phytochemical and matrix components, the multivariate analysis indicated that glycemic response was associated with the overall compositional profile of the hybrids rather than with a single constituent. These findings highlight the importance of considering the whole-grain matrix when assessing popcorn quality and its potential relationship with glycemic response. Popcorn hybrids, particularly Popcorn 2, demonstrate potential as functional maize for developing nutritionally enriched snack foods with higher levels of bioactive compounds and a better GI profile. These results encourage further examination and selection of maize germplasm focusing on combined nutritional and phytochemical characteristics to enhance popcorn’s functional benefits.

## Figures and Tables

**Table 1:** Estimated glycemic index of popcorn hybrids.

Sample	Estimated Glycemic Index (eGI)
Popcorn C	67.41 ± 0.37 a
Popcorn 1	48.37 ± 0.38 b
Popcorn 2	45.64 ± 0.02 c
Popcorn 3	47.82 ± 0.24 b
Popcorn 4	46.27 ± 0.18 c
Popcorn 5	46.13 ± 0.16 c

The results are expressed as the means of duplicates ± SD (standard deviation). Different letters per column indicate significant differences (*p* < 0.05) by the Tukey test.

## Data Availability

The original contributions presented in this study are included in the article/Supplementary Materials. Further inquiries can be directed to the corresponding authors.

## Supplementary Materials

The following supporting information can be downloaded at https://www.mdpi.com/article/10.3390/foods15183333/s1, Table S1: Chromatographic and mass spectrometric characteristics of free phenolic acids, bound phenolic acids, and hydroxycinnamic acid amides identified in fractions of grain and popcorn samples by UPLC-PDA-QDA-ESI+; Table S2: Content of free phenolic acids in grain samples; Table S3: Content of free phenolic acids in popcorn samples; Table S4: Content of bound phenolic acids in grain samples; Table S5: Content of bound phenolic acids in different popcorn samples; Table S6: Phytosterols quantification in grains and popcorn samples analyzed by UPLC-PDA-ELSD; Table S7. Pearson and Spearman correlation analyses of phytochemical composition and eGI of popcorn samples; Figure S1: Release of glucose over time of different popcorn hybrid samples; Figure S2: Combined PCA of the harmonized phenolic profile of raw grain (circles) and popped product (triangles); arrows connect the raw and popped centroids of each genotype × fraction combination, depicting the processing trajectory.

## Abbreviations

The following abbreviations are used in this manuscript:

5,5′-DFA	5,5′-Diferulic acid
8-O-4′-DFA	8-O-4′-Diferulic Acid
8,5′-DFA BZF	8,5′-Diferulic Acid Benzofuran Form
8,5′-DFA OPC	8,5′-Diferulic Acid Open Form
8,8′-DFA	8,8′-Diferulic Acid
ABTS	2,2′-Azino-Bis(3-Ethylbenzothiazolin)-6-Sulfonic Acid
AUC	Area Under the Curve
c-CA	Cis-p-Coumaric Acid
DCP	Dicoumaroylputrescine
DFA	Diferulic Acid
DFP	DiferuloylPutrescine
DFP-1	Diferuloylputrescine Isomer 1
DFP-2	Diferoylputrescine Isomer 2
DFP-3	Diferuloylputrescine Isomer 3
DFP-4	Diferuloylputrescine Isomer 4
DPPH	2,2-diphenyl-1-picrylhydrazyl
eGI	Estimated Glycemic Index
EtOH	Ethanol
FA	Ferulic Acid
FCP	N-feruloyl-N’-coumaroylputrescine
FP	N-feruloylputrescine
FPN	Feruloylputrescine
GI	Glycemic Index
GOPOD	Glucose Oxidase-Peroxidase
HCAAs	Hydroxycinnamic Acid Amides
HI	Hydrolysis Index
HCl	Hydrochloric Acid
IDF	Insoluble Dietary Fiber
INIFAP	Instituto Nacional de Investigaciones Forestales, Agrícolas y Pecuarias
K_2_S_2_O_8_	Potassium Persulfate
NaOH	Sodium Hydroxide
OPLS-DA	Orthogonal Partial Least Squares Discriminant Analysis
PCA	Principal Component Analysis
p-CA	p-Coumaric Acid
PLS-DA	Partial Least Squares Discriminant Analysis
PES	Polyethersulfone
t-CA	Trans-p-Coumaric Acid
SD	Standard Deviation
SDF	Soluble Dietary Fiber
TDF	Total Dietary Fiber
UPLC-PDA-ELSD	Ultra-Performance Liquid Chromatography Coupled with a Photodiode Array Detector
UPLC-PDA-QDA-ESI	Ultra-Performance Liquid Chromatography-Photodiode Array-Quadrupole Detector Accuracy-Electrospray Ionization
VIP	Variable Importance in Projection
