# Supplementary material for: Unraveling the Food Matrix Components Underlying Glycemic Response in Yellow Popcorn Hybrids (Zea mays var. ‘Everta’)
Source: Foods. 2026 Sep 20;15(18):3333. doi: 10.3390/foods15183333 (PMC13606733; doi:10.3390/foods15183333)
Supplement: Supplementary file 1 [file foods-15-03333-s001.zip › foods-4547589-supplementary.pdf]

# Unraveling the food matrix components underlying glycemic response in yellow popcorn (*Zea mays* var. 'Everta') hybrids

**Emiko Daniela González-Nimi[1], Janet A. Gutiérrez-Uribe[1*], Benjamín Vázquez-Rodríguez[1,2], Marilena Antunes-Ricardo[1,2], Ricardo Ernesto Preciado-Ortiz[3] and Anayansi Escalante-Aburto[1,2,*]**

[1] Tecnologico de Monterrey, School of Engineering and Sciences, Eugenio Garza Sada 2501, Monterrey, N. L. 64849, Mexico.
[2] Tecnologico de Monterrey, Institute for Obesity Research, Av. Eugenio Garza Sada 2501, Monterrey, N. L. 64849, Mexico.
[3] Programa de Mejoramiento Genético de Maíz, Campo Experimental del Bajío, Instituto Nacional de Investigaciones Forestales, Agrícolas y Pecuarias (INIFAP), Carretera Celaya-San Miguel de Allende km 6.5, Celaya, Guanajuato, 38110, Mexico.
* Correspondence: anayansi.escalante@tec.mx; jagu@tec.mx

**Table S1.** Chromatographic and mass spectrometric characteristics of free phenolic acids, bound phenolic acids, and hydroxycinnamic acid amides identified in fractions of grain and popcorn samples by UPLC-PDA-QDA-ESI +.

| Sample | Peak | Proposed compound | [M+H]+ (m/z) | MS Fragments/Adducts (m/z) | UV max (λmax nm) | Reference |
|---|---|---|---|---|---|---|
| *Free phenolic compounds* | | | | | | |
| Grain | 1 | p-Coumaric acid (p-CA) | 165.2 | 184.5, 206.4 | 309 | (Bento-Silva et al., 2020) |
| | 2 | Ferulic acid (FA) | 195.2 | 236.2, 275.8 | 327.6 | (Bento-Silva et al., 2020) |
| | 3 | Feruloylputrescine (FP) | 265.2 | 263.4, 181.2 | ~293 – 320 | (Li et al., 2018) |
| | 4 | Di-p-coumaroylputrescine (DCP) | 381.3 | 261.3, 139.1 | ~305 – 312 | (Li et al., 2018) |
| | 5 | N-feruloyl-N'-coumaroylputrescine (FCP) | 411.3 | 314.4 | ~293 – 320 | (Bento-Silva et al., 2020) (Li et al., 2018) |
| | 6 | Diferuloylputrescine (DFP) | 441.5 | 140.1 | ~293 – 325 | (Li et al., 2018) |
| Popcorn | 1 | Cis-p-Coumaric acid (c-CA) | 165.3 | 184.5, 206.4 | 309 | (Bento-Silva et al., 2020) |
| | 2 | Trans-p-Coumaric acid (t-CA) | 165.2 | 184.5, 206.4 | 309 | (Bento-Silva et al., 2020) |
| | 3 | Ferulic acid (FA) | 195.2 | 236.2, 275.8 | 327.6 | (Bento-Silva et al., 2020) |
| | 4 | N-feruloylputrescine (FP) | 265.2 | 263.4, 181.2 | 293 – 320 | (Li et al., 2018) |
| | 5 | Diferuloylputrescine Isomer 1 (DFP-1) | 441.4 | 265.2, 140.1 | 293.6, 316.4 | (Li et al., 2018) |
| | 6 | Diferuloylputrescine Isomer 2 (DFP-2) | 441.4 | 265.2, 261.2 | 291.7 | (Li et al., 2018) |
| | 7 | N-feruloyl-N'-coumaroylputrescine (FCP)* | 411.3 | 314.4 | ~292.3, 311.5 | (Bento-Silva et al., 2020) (Li et al., 2018) |
| | 8 | Diferuloylputrescine Isomer 3 (DFP-3)* | 441.4 | 265.2 | 292.3, 311.5 | (Li et al., 2018) |
| | 9 | Diferuloylputrescine Isomer 4 (DFP-4) | 441.5 | 265.3, 442.5 | 293.0, 317.1 | (Li et al., 2018) |
| *Bound phenolic compounds* | | | | | | |
| Grain | 1 | p-Coumaric acid (p-CA) | 165.2 | 184.5, 206.4 | 309 | (Chen et al., 2021) |

| | | | | | | |
|---|---|---|---|---|---|---|
| | 2 | Ferulic acid (FA) | 195.2 | 236.2, 275.8 | 327.6 | (Menga et al., 2020) |
| | 3 | 8,5'-Diferulic acid open form (8,5'-DFA OPC) | 387.3 | 275.0, 283.4 | *322.6* | (Bento-Silva et al., 2020) |
| | 4 | 5,5'-Diferulic acid (5,5'-DFA) | 387.4 | 351.2, 507.5 | *323.9* | (Calloni et al., 2021) |
| | 5 | N-feruloyl-N'-coumaroylputrescine (FCP) | 265.2 | 263.4, 181.2 | ~293 – 320 | (Roumani et al., 2021) |
| | 6 | Diferuloylputrescine (DFP) | 381.3 | 261.3, 139.1 | ~305 – 312 | (Ugena et al., 2022) |
| | 7 | 8-O-4'-Diferulic acid (8-O-4'-DFA) | 387.3 | 274.8, 369.5 | 325.1 | (Liu et al., 2023) |
| | 8 | 8,5'-Diferulic acid benzofuran form (8,5'-DFA BZF) | 387.4 | 233.2, 463.3 | 323.9 | (Ni et al., 2020) |
| | 9 | 8,8'-Diferulic acid (8,8'-DFA) | 387.3 | 343.3, 579.2 | 321.4 | (Zheng et al., 2021) |
| Popcorn | 1 | p-Coumaric acid (p-CA) | 165.2 | 184.5, 206.4 | 309 | (Bento-Silva et al., 2020) |
| | 2 | Ferulic acid (FA) | 195.2 | 236.2, 275.8 | 327.6 | (Bento-Silva et al., 2020) |
| | 3 | 8,5'-Diferulic acid open form (8,5'-DFA OPC) | 387.3 | 275.0, 283.4 | 322.6 | (Bento-Silva et al., 2020) |
| | 4 | N-feruloylputrescine (FP) | 265.2 | 263.4, 181.2 | 293 – 320 | (Li et al., 2018) |
| | 5 | 5,5'-Diferulic acid (5,5'-DFA) | 387.4 | 351.2, 507.5 | 323.9 | (Calloni et al., 2021) |
| | 6 | Di-p-coumaroylputrescine (DCP) | 381.3 | 261.3, 139.1 | ~305 – 312 | (Li et al., 2018) |
| | 7 | N-feruloyl-N'-coumaroylputrescine (FCP) | 411.3 | 314.4 | ~292.3, 311.5 | (Bento-Silva et al., 2020) (Li et al., 2018) |
| | 8 | Diferuloylputrescine (DFP) | 381.3 | 261.3, 139.1 | ~305 – 312 | (Ugena et al., 2022) |
| | 9 | 8-O-4'-Diferulic acid (8-O-4'-DFA) | 387.3 | 274.8, 369.5 | 325.1 | (Liu et al., 2023) |
| | 10 | 8,5'-Diferulic acid benzofuran form (8,5'-DFA BZF) | 387.4 | 233.2, 463.3 | 323.9 | (Ni et al., 2020) |
| | 11 | 8,8'-Diferulic acid (8,8'-DFA) | 387.3 | 343.3, 579.2 | 321.4 | (Zheng et al., 2021) |

*Chromatographic data indicate a co-elution event between FCP (m/z 411) and DFP-3 isomer signal (m/z 441) in free phenolic compounds of popcorn.

**Table S2.** Content of free phenolic acids in grain samples.

| Sample | Fraction | p-CA | FA | FP | DCP | FCP | DFP |
|---|---|---|---|---|---|---|---|
| Grain C | Fine | 73.3 ± 2.0$^{a}$ | 9.6 ± 0.2$^{ab}$ | n.d. | n.d. | 10.2 ± 0.5$^{cdef}$ | 57.8 ± 3.0$^{b}$ |
| | Coarse | 64.0 ± 2.3$^{ab}$ | 6.8 ± 0.6$^{cdef}$ | n.d. | n.d. | 14.5 ± 0.3$^{a}$ | 99.0 ± 2.2$^{a}$ |
| Grain 1 | Fine | 46.8 ± 0.4$^{cd}$ | 8.8 ± 0.7$^{abcd}$ | 8.9 ± 0.04$^{abc}$ | 5.3 ± 0.22$^{a}$ | 6.5 ± 0.3$^{gh}$ | 17.3 ± 1.6$^{fg}$ |
| | Coarse | 39.8 ± 0.1$^{d}$ | 7.3 ± 0.1$^{bcdef}$ | 7.1 ± 1.09$^{abc}$ | 6.7 ± 0.31$^{a}$ | 8.2 ± 1.2$^{fg}$ | 33.2 ± 0.3$^{cd}$ |
| Grain 2 | Fine | 54.6 ± 5.3$^{bc}$ | 9.3 ± 0.2$^{abc}$ | 10.1 ± 0.60$^{a}$ | 5.4 ± 0.77$^{a}$ | 9.7 ± 0.9$^{def}$ | 28.2 ± 2.9$^{de}$ |
| | Coarse | 46.2 ± 0.5$^{cd}$ | 7.8 ± 1.0$^{abcdef}$ | 8.2 ± 0.56$^{abc}$ | 6.6 ± 0.77$^{a}$ | 13.2 ± 0.6$^{ab}$ | 41.1 ± 2.2$^{c}$ |
| Grain 3 | Fine | 52.9 ± 6.4$^{cd}$ | 10.3 ± 0.8$^{a}$ | n.d. | n.d. | 10.9 ± 0.8$^{bcde}$ | 34.9 ± 4.6$^{cd}$ |
| | Coarse | 29.3 ± 3.5$^{e}$ | 6.3 ± 0.3$^{def}$ | 6.1 ± 0.54$^{bc}$ | 5.6 ± 0.54$^{a}$ | 11.9 ± 1.0$^{bcd}$ | 54.5 ± 2.0$^{b}$ |
| Grain 4 | Fine | 61.5 ± 2.8$^{b}$ | 8.4 ± 0.4$^{abcde}$ | 7.2 ± 2.24$^{abc}$ | n.d. | 7.1 ± 0.0$^{gh}$ | 23.4 ± 0.5$^{ef}$ |
| | Coarse | 42.4 ± 3.5$^{d}$ | 5.6 ± 0.4$^{f}$ | 7.2 ± 0.62$^{abc}$ | 5.4 ± 0.03$^{a}$ | 9.0 ± 0.6$^{efg}$ | 33.7 ± 2.0$^{cd}$ |
| Grain 5 | Fine | 74.0 ± 3.3$^{a}$ | 9.9 ± 0.3$^{ab}$ | 5.7 ± 0.10$^{c}$ | 5.7 ± 0.03$^{a}$ | 5.4 ± 0.1$^{h}$ | 14.4 ± 0.8$^{g}$ |
| | Coarse | 55.4 ± 1.4$^{bc}$ | 6.0 ± 1.6$^{ef}$ | 9.0 ± 0.84$^{ab}$ | 6.6 ± 0.53$^{a}$ | 12.4 ± 0.2$^{abc}$ | 18.2 ± 1.0$^{fg}$ |

Values of p-coumaric acid expressed as μg coumaric acid/g maize, values of FA, FP, DCP, FCP and DFP expressed as μg ferulic acid/g maize. p-CA = para-coumaric acid, FA = Ferulic acid, FP = Feruloylputrescine; DCP = Dicoumaroylputrescine; FCP = N-feruloyl-N'-coumaroylputrescine; DFP = Diferuloylputrescine; n.d. = Non detected. Data are presented as means ± standard deviation. Different superscript letters within the same column indicate significant differences among interactions (Sample × Fraction) according to Tukey-Kramer's multiple comparison test ($p < 0.05$).

**Table S3.** Content of free phenolic acids in popcorn samples.

| Sample | Fraction | c-CA | t-CA | FA | FP | DFP-1 | DFP -2 | FCP +DFP-3 | DFP-4 |
|---|---|---|---|---|---|---|---|---|---|
| Popcorn C | Fine | $2.8 \pm 0.2^{cd}$ | $5.7 \pm 0.1^{a}$ | $1.19 \pm 0.01^{bc}$ | n.d. | n.d. | $17.9 \pm 0.3^{a}$ | $4.5 \pm 0.05^{b}$ | $18.1 \pm 0.2^{a}$ |
| | Coarse | $1.1 \pm 0.1^{f}$ | $1.22 \pm 0.08^{cde}$ | $1.39 \pm 0.01^{b}$ | n.d. | n.d. | 0.69 ± 01 | $11.6 \pm 0.02^{a}$ | 13.5 ± 0.1b |
| Popcorn 1 | Fine | $4.2 \pm 0.3^{ab}$ | $2.3 \pm 0.2^{b}$ | $1.3 \pm 0.1^{bc}$ | $2.5 \pm 0.2^{b}$ | $9.6 \pm 0.4^{a}$ | 9.1 ± 0.6 | $5.1 \pm 0.3^{b}$ | $15.9 \pm 1.1^{ab}$ |
| | Coarse | $3.1 \pm 0.4^{bc}$ | n.d. | $0.77 \pm 0.03^{bc}$ | $0.41 \pm 0.01^{c}$ | $1.4 \pm 0.1^{efg}$ | 3.4 ± 0.3 | $1.6 \pm 0.2d^{e}$ | $4.9 \pm 0.6^{e}$ |
| Popcorn 2 | Fine | $1.9 \pm 0.2^{def}$ | $1.4 \pm 0.2^{cd}$ | $1.0 \pm 0.1^{bc}$ | $3.4 \pm 0.5^{a}$ | $3.5 \pm 0.5^{cd}$ | 5.0 ± 1.2 | $3.1 \pm 0.6^{c}$ | $7.6 \pm 1.3^{cd}$ |
| | Coarse | $1.2 \pm 0.2^{f}$ | $0.71 \pm 0.06^{def}$ | $0.7 \pm 0.1^{c}$ | n.d. | $2.9 \pm 0.1^{cde}$ | 3.7 ± 0.3 | $1.77 \pm 0.03^{de}$ | $5.3 \pm 0.2^{de}$ |
| Popcorn 3 | Fine | $2.4 \pm 0.6^{cde}$ | $2.5 \pm 0.5^{b}$ | $1.3 \pm 0.2b^{c}$ | n.d. | $10.5 \pm 1.0^{a}$ | 10.6 ± 0.4 | $5.2 \pm 0.3^{b}$ | $18.1 \pm 0.3^{a}$ |
| | Coarse | $1.3 \pm 0.1^{ef}$ | $1.19 \pm 0.01^{cde}$ | $1.2 \pm 0.0^{bc}$ | n.d. | $5.5 \pm 0.3^{b}$ | 5.3 ± 0.6 | $2.4 \pm 0.3^{cd}$ | $8.0 \pm 1.1^{c}$ |
| Popcorn 4 | Fine | $3.3 \pm 0.3^{bc}$ | $1.9 \pm 0.2^{bc}$ | $2.3 \pm 0.4^{a}$ | $3.9 \pm 0.4^{a}$ | $4.2 \pm 0.6^{bc}$ | 6.2 ± 0.3 | $3.2 \pm 0.2^{c}$ | $9.7 \pm 0.6^{c}$ |
| | Coarse | $1.7 \pm 0.1^{def}$ | $0.4 \pm 0.28^{ef}$ | $0.8 \pm 0.1^{bc}$ | $0.38 \pm 0.02^{c}$ | $1.5 \pm 0.2^{efg}$ | 0.8 ± 0.1 | $1.1 \pm 0.1^{ef}$ | $3.2 \pm 0.4^{ef}$ |
| Popcorn 5 | Fine | $4.7 \pm 0.2^{a}$ | n.d. | $1.4 \pm 0.3^{b}$ | $0.71 \pm 0.01^{c}$ | $2.4 \pm 0.1^{def}$ | 3.4 ± 0.3 | $1.4 \pm 0.03^{e}$ | $3.1 \pm 0.05^{ef}$ |
| | Coarse | $3.4 \pm 0.4^{bc}$ | n.d. | $1.0 \pm 0.2^{bc}$ | $0.51 \pm 0.02^{c}$ | $1.1 \pm 0.2^{fg}$ | 1.0 ± 0.2 | $0.3 \pm 0.05^{f}$ | $1.3 \pm 0.33^{f}$ |

Values of CA expressed as µg CA/g maize, values of FA, DFA, FCP, and DFP expressed as µg ferulic acid equivalents/g popcorn.; values of FA, FP, DFP and FCP, expressed as mg ferulic acid equivalents/g popcorn. Data are presented as means. c-CA = cis-Coumaric acid; t-CA = trans-Coumaric acid; FA = Ferulic acid; FP = N-feruloylputrescine; DFP = Diferuloylputrescine; FCP = N-feruloyl-N'-coumaroylputrescine; Lp = Light phase; Cp = coarse phase; ND = Not detected. FCP and DFP-3 co-eluted and were quantified as a single combined peak (FCP + DFP-3). Data are presented as means ± standard deviation. Different superscript letters within the same column indicate significant differences among interactions (Sample × Fraction) according to Tukey-Kramer's multiple comparison test ($p < 0.05$).

**Table S4.** Content of bound phenolic acids in grain samples.

| Sample | Fraction | p-CA | FA | 8,5'-DFA OPC | 5,5'-DFA | FCP | DFP | 8-O-4'-DFA | 8,5'-DFA BZF | 8,8'-DFA |
|---|---|---|---|---|---|---|---|---|---|---|
| Grain C | Fine | 90.40 ± 3.10$^{cde}$ | 1191.70 ± 45.20$^{ef}$ | 58.30 ± 3.20$^{cd}$ | 104.00 ± 1.10$^{bc}$ | 58.30 ± 1.10$^{a}$ | 29.6 ± 0.5$^{bc}$ | 111.80 ± 0.91$^{c}$ | 38.40 ± 2.60$^{cd}$ | 37.30 ± 0.59$^{d}$ |
| | Coarse | 53.10 ± 10.00$^{de}$ | 684.10 ± 138.30$^{fg}$ | 26.50 ± 2.60$^{ef}$ | 111.30 ± 67.40$^{bc}$ | 16.10 ± 9.09$^{bcd}$ | 55.60 ± 4.38$^{a}$ | 174.30 ± 5.39$^{b}$ | 34.00 ± 5.63$^{d}$ | 56.70 ± 3.18$^{bc}$ |
| Grain 1 | Fine | 143.00 ± 13.09$^{abcd}$ | 1394.90 ± 146.10$^{de}$ | 56.00 ± 6.51$^{cd}$ | 111.30 ± 14.59$^{bc}$ | 9.80 ± 9.09$^{bcd}$ | 18.10 ± 0.8$^{cde}$ | 119.20 ± 12.70$^{c}$ | 54.40 ± 1.40$^{b}$ | 36.70 ± 3.39$^{d}$ |
| | Coarse | 186.90 ± 14.59$^{abc}$ | 2439.20 ± 235.00$^{b}$ | 93.10 ± 2.60$^{ab}$ | 160.60 ± 20.51$^{ab}$ | 16.10 ± 1.60$^{bcd}$ | 55.90 ± 9.50$^{a}$ | 178.80 ± 24.59$^{b}$ | 45.40 ± 5.20$^{bcd}$ | 57.90 ± 1.15$^{abc}$ |
| Grain 2 | Fine | 29.50 ± 3.00$^{e}$ | 391.70 ± 49.40$^{g}$ | 16.00 ± 1.70 | 31.70 ± 4.10$^{c}$ | 2.70 ± 2.70$^{cd}$ | 5.70 ± 0.40$^{ef}$ | 31.10 ± 4.10$^{d}$ | 10.80 ± 0.10$^{e}$ | 10.10 ± 1.30$^{e}$ |
| | Coarse | 237.30 ± 22.80$^{a}$ | 3074.70 ± 35.30$^{a}$ | 103.80 ± 7.50$^{a}$ | 225.80 ± 1.40$^{a}$ | 29.60 ± 0.49$^{b}$ | 35.70 ± 0.69$^{b}$ | 222.20 ± 3.10$^{a}$ | 83.60 ± 1.70$^{a}$ | 63.40 ± 1.99$^{ab}$ |
| Grain 3 | Fine | 114.70 ± 1.50$^{bcde}$ | 1034.40 ± 15.90$^{ef}$ | 49.50 ± 0.10$^{de}$ | 93.60 ± 4.50$^{bc}$ | 21.50 ± 0.69$^{bc}$ | 33.60 ± 0.66$^{b}$ | 89.80 ± 4.50$^{c}$ | 36.90 ± 0.91$^{d}$ | 32.80 ± 2.40$^{d}$ |
| | Coarse | 161.70 ± 84.20$^{abc}$ | 2030.40 ± 54.20$^{bc}$ | 78.10 ± 0.20$^{bc}$ | 177.50 ± 6.70$^{ab}$ | 22.40 ± 4.89$^{bc}$ | 28.00 ± 2.14$^{bcd}$ | 189.30 ± 1.00$^{ab}$ | 44.40 ± 5.60$^{bcd}$ | 52.90 ± 1.50$^{c}$ |
| Grain 4 | Fine | 111.70 ± 13.10$^{bcde}$ | 1291.20 ± 173.90$^{e}$ | 52.60 ± 6.60$^{d}$ | 96.80 ± 17.90$^{bc}$ | 10.10 ± 10.10$^{bcd}$ | 15.70 ± 3.63$^{de}$ | 93.80 ± 19.20$^{c}$ | 44.60 ± 0.78$^{bcd}$ | 36.40 ± 1.77$^{d}$ |
| | Coarse | 203.00 ± 11.70$^{ab}$ | 1952.10 ± 357.80$^{bcd}$ | 78.30 ± 17.20$^{bc}$ | 170.30 ± 34.90$^{ab}$ | 21.80 ± 2.90$^{bc}$ | 25.20 ± 2.09$^{bcd}$ | 198.60 ± 0.65$^{ab}$ | 44.80 ± 6.29$^{bcd}$ | 65.40 ± 1.84$^{a}$ |
| Grain 5 | Fine | 89.70 ± 0.59$^{cde}$ | 1160.50 ± 6.19$^{ef}$ | 55.90 ± 0.20 | 115.10 ± 0.69$^{bc}$ | n.d. | n.d. | 109.60 ± 4.79$^{c}$ | 92.00 ± 0.65$^{a}$ | 39.10 ± 2.09$^{d}$ |
| | Coarse | 114.40 ± 5.50$^{bcde}$ | 1446.70 ± 75.10$^{cde}$ | 60.50 ± 4.20$^{cd}$ | 127.90 ± 2.60$^{b}$ | 18.90 ± 0.69$^{bcd}$ | 16.30 ± 3.37$^{cde}$ | 122.90 ± 1.40$^{c}$ | 51.00 ± 1.13$^{bc}$ | 40.14 ± 0.69$^{d}$ |

Values of CA expressed as µg CA/g grain, values of FA, DFA, FCP and DFP expressed as µg ferulic acid equivalents/g grain. p-CA = para-coumaric acid, FA = Ferulic acid, DFA = Diferulic acid; FCP = N-feruloyl-N'-coumaroylputrescine; DFP = Diferuloylputrescine; OPC open circle; ND = Non detected. Data are presented as means ± standard deviation. Different superscript letters within the same column indicate significant differences among interactions (Sample × Fraction) according to Tukey-Kramer’s multiple comparison test ($p < 0.05$)

**Table S5.** Content of bound phenolic acids in different popcorn samples.

| Sample | Fraction | p-CA | FA | 8,5'-DFA OPC | FP | 5,5'-DFA | DCP | FCP | DFP | 8-O-4'-DFA | 8,5'-DFA BZF | 8,8'-DFA |
|---|---|---|---|---|---|---|---|---|---|---|---|---|
| Popcorn C | Fine | 469.89 ± 23.33$^{a}$ | 6,764.94 ± 203.84$^{a}$ | 276.09 ± 10.95$^{b}$ | 102.35 ± 2.00$^{a}$ | 722.33 ± 60.84$^{a}$ | 39.46 ± 19.73$^{a}$ | 79.39 ± 1.50$^{a}$ | 194.68 ± 14.06$^{a}$ | 798.58 ± 59.29$^{a}$ | 266.03 ± 15.14$^{a}$ | 238.12 ± 10.40$^{a}$ |
| | Coarse | 152.70 ± 38.27$^{de}$ | 1,695.62 ± 267.52$^{ef}$ | 73.81 ± 0.28$^{e}$ | 33.23 ± 7.29$^{d}$ | 139.98 ± 5.70$^{de}$ | n.d. | 10.43 ± 10.43$^{cd}$ | 44.93 ± 13.34$^{b}$ | 162.74 ± 6.26$^{efg}$ | 65.10 ± 8.65$^{bcd}$ | 53.57 ± 6.67$^{de}$ |
| Popcorn 1 | Fine | 386.11 ± 10.38$^{b}$ | 3,881.60 ± 267.52$^{b}$ | 355.80 ± 5.70$^{a}$ | 67.39 ± 5.89$^{b}$ | 355.80 ± 8.53$^{b}$ | n.d. | 43.80 ± 0.22$^{b}$ | 60.85 ± 0.88$^{b}$ | 375.55 ± 9.84$^{b}$ | 96.14 ± 20.84$^{b}$ | 121.38 ± 3.13$^{b}$ |
| | Coarse | 159.45 ± 7.94$^{de}$ | 1,747.90 ± 474.39$^{ef}$ | 73.49 ± 3.02$^{e}$ | 35.55 ± 0.00$^{d}$ | 122.02 ± 5.62$^{de}$ | n.d. | 8.35 ± 8.35$^{cd}$ | 43.03 ± 4.54$^{b}$ | 133.97 ± 5.78$^{fg}$ | 41.95 ± 0.07$^{cde}$ | 43.02 ± 14.04$^{e}$ |
| Popcorn 2 | Fine | 297.87 ± 44.55$^{c}$ | 3,410.45 ± 525.66$^{bc}$ | 173.01 ± 34.10$^{cd}$ | 52.73 ± 14.43$^{bcd}$ | 275.41 ± 48.04$^{bc}$ | 5.54 ± 5.54$^{b}$ | 43.25 ± 12.47$^{b}$ | 43.41 ± 13.53$^{b}$ | 269.48 ± 50.90$^{cd}$ | 55.61 ± 7.60$^{cd}$ | 85.06 ± 22.27$^{bcd}$ |
| | Coarse | 51.25 ± 0.22$^{f}$ | 800.37 ± 4.53$^{f}$ | 49.04 ± 0.35$^{e}$ | n.d. | 83.24 ± 1.13$^{e}$ | n.d. | n.d. | 46.24 ± 3.15$^{b}$ | 78.06 ± 10.93$^{g}$ | 37.96 ± 1.09$^{de}$ | 44.99 ± 1.29$^{e}$ |
| Popcorn 3 | Fine | 279.25 ± 13.10$^{c}$ | 3,949.12 ± 85.93$^{b}$ | 207.79 ± 4.52$^{cd}$ | 67.39 ± 1.47$^{b}$ | 324.52 ± 7.06$^{b}$ | n.d. | 43.39 ± 0.94$^{b}$ | 57.13 ± 1.24$^{b}$ | 321.44 ± 6.99$^{bc}$ | 63.43 ± 1.38$^{bcd}$ | 107.68 ± 2.34$^{bc}$ |
| | Coarse | 149.15 ± 0.55$^{de}$ | 1,376.01 ± 8.39$^{ef}$ | 73.02 ± 22.65$^{e}$ | 45.09 ± 0.00$^{cd}$ | 113.80 ± 28.84$^{de}$ | n.d. | n.d. | 42.26 ± 2.32$^{b}$ | 118.52 ± 26.45$^{fg}$ | 44.41 ± 2.58$^{cde}$ | 53.91 ± 8.69$^{de}$ |
| Popcorn 4 | Fine | 323.63 ± 19.75$^{bc}$ | 3,777.60 ± 122.16$^{bc}$ | 214.46 ± 2.03$^{c}$ | 72.35 ± 1.97$^{b}$ | 362.50 ± 31.20$^{b}$ | n.d. | 34.87 ± 0.00$^{b}$ | 43.56 ± 4.55$^{b}$ | 360.61 ± 26.88$^{bc}$ | 62.31 ± 0.76$^{cd}$ | 117.23 ± 0.61$^{b}$ |
| | Coarse | 88.95 ± 4.20$^{ef}$ | 932.06 ± 33.51$^{ef}$ | 59.97 ± 5.33$^{e}$ | n.d. | 90.37 ± 8.62$^{e}$ | n.d. | n.d. | 38.32 ± 0.13$^{bc}$ | 103.21 ± 0.91$^{fg}$ | 46.31 ± 1.89$^{cde}$ | 48.43 ± 3.83$^{de}$ |
| Popcorn 5 | Fine | 192.81 ± 2.65$^{d}$ | 2,819.51 ± 202.94$^{cd}$ | 156.98 ± 0.82$^{d}$ | 58.00 ± 5.22$^{bc}$ | 277.94 ± 13.67$^{bc}$ | 8.95 ± 0.54$^{b}$ | 30.66 ± 7.54$^{bc}$ | 11.58 ± 0.58$^{c}$ | 259.72 ± 10.18$^{def}$ | 19.67 ± 1.57$^{e}$ | 100.41 ± 12.98$^{bc}$ |
| | Coarse | 155.81 ± 4.201$^{de}$ | 1910.40 ± 97.80$^{de}$ | 59.97 ± 17.54$^{e}$ | 45.94 ± 3.79$^{cd}$ | 195.52 ± 10.42$^{cd}$ | n.d. | n.d. | 40.98 ± 2.78$^{b}$ | 181.12 ± 9.65$^{c}$ | 73.18 ± 4.02$^{bc}$ | 72.23 ± 3.83$^{cde}$ |

Values of CA expressed as μg CA/g maize, values of FA, FP, DCP ,DFA, FCP and DFP expressed as μg ferulic acid equivalents/g maize. CA = Coumaric acid, FA = Ferulic acid, FP = Feruloylputrescine, DCP = Dicoumaroylputrescine, DFA = Diferulic acid; FCP = N-feruloyl-N'-coumaroylputrescine; DFP =Diferuloylputrescine; OPC open circle; n.d. = Not detected. Data are presented as means ± standard deviation. Different superscript letters within the same column indicate significant differences among interactions (Sample × Fraction) according to Tukey-Kramer's multiple comparison test ($p < 0.05$).

**Table S6**. Phytosterols quantification in grains and popcorn samples analyzed by UPLC-PDA-ELSD

| Sample | Fraction | Stigmasterol | Campesterol | β-Sitosterol | Total Phytosterols |
|---|---|---|---|---|---|
| Grain C | Fine | 56.37 ± 2.57 [a] | n.d. | 62.24 ± 3.46 [d-f] | 118.62 ± 6.02 [e-g] |
| | Coarse | 17.63 ± 1.98 [f-j] | n.d. | 17.62 ± 2.69 [jk] | 35.25 ± 4.67 [k-m] |
| Grain 1 | Fine | 25.33 ± 1.90 [de] | n.d. | 32.53 ± 2.73 [h-j] | 57.86 ± 4.63 [i-l] |
| | Coarse | 12.48 ± 0.09 [i-j] | n.d. | 12.77 ± 0.35 [k] | 25.24 ± 0.43 [lm] |
| Grain 2 | Fine | 33.50 ± 0.66 [bc] | n.d. | 38.20 ± 1.49 [h-i] | 71.70 ± 2.15 [h-j] |
| | Coarse | 13.80 ± 1.43 [h-j] | n.d. | 13.39 ± 1.82 [k] | 27.19 ± 3.26 [lm] |
| Grain 3 | Fine | 34.45 ± 1.67 [bc] | n.d. | 40.66 ± 2.24 [g-i] | 75.10 ± 3.91 [h-j] |
| | Coarse | 23.84 ± 1.28 [d-f] | n.d. | 28.44 ± 1.85 [i-k] | 52.28 ± 3.13 [j-m] |
| Grain 4 | Fine | 29.44 ± 0.59 [cd] | n.d. | 33.82 ± 0.86 [h-j] | 63.26 ± 1.45 [h-k] |
| | Coarse | 25.14 ± 1.86 [de] | n.d. | 29.40 ± 2.30 [i-k] | 54.54 ± 4.16 [i-m] |
| Grain 5 | Fine | 39.32 ± 0.96 [b] | n.d. | 48.48 ± 4.50 [f-h] | 87.80 ± 5.45 [g-i] |
| | Coarse | 23.90 ± 1.01 [d-f] | n.d. | 26.93 ± 0.61 [i-k] | 50.83 ± 1.63 [j-m] |
| Popcorn C | Fine | 16.79 ± 0.19 [g-j] | 9.61 ± 0.42 [e] | 69.94 ± 2.23 [c-e] | 96.33 ± 2.85 [f-h] |
| | Coarse | 21.13 ± 5.55 [e-g] | n.d. | n.d. | 21.13 ± 5.55 [m] |
| Popcorn 1 | Fine | 29.42 ± 0.14 [cd] | 23.23 ± 0.66 [de] | 121.05 ± 5.44 [a] | 173.70 ± 4.92 [ab] |
| | Coarse | 19.33 ± 0.35 [e-i] | 11.27 ± 0.76 [e] | 63.63 ± 4.51 [d-f] | 94.23 ± 4.92 [f-h] |
| Popcorn 2 | Fine | 17.99 ± 0.47 [f-j] | 43.58 ± 2.19 [b-d] | 96.34 ± 8.48 [b] | 157.92 ± 11.14 [b-d] |
| | Coarse | 14.00 ± 0.05 [h-j] | 10.75 ± 0.20 [e] | 56.47 ± 1.31 [e-g] | 81.22 ± 1.46 [h-j] |
| Popcorn 3 | Fine | 20.40 ± 0.84 [e-h] | 61.53 ± 0.69 [ab] | 91.85 ± 3.35 [b] | 173.77 ± 3.20 [ab] |
| | Coarse | 17.96 ± 2.11 [f-j] | 41.91 ± 7.43 [b-d] | 79.67 ± 11.88 [b-d] | 139.55 ± 21.42 [c-e] |
| Popcorn 4 | Fine | 19.21 ± 2.57 [e.i] | 57.48 ± 10.42 [a-c] | 88.66 ± 0.20 [b] | 165.35 ± 12.79 [a-c] |
| | Coarse | 12.05 ± 0.26 [j] | 29.09 ± 3.01 [c-e] | 86.66 ± 9.00 [bc] | 127.80 ± 12.28 [d-f] |
| Popcorn 5 | Fine | 20.52 ± 0.26 [e-h] | 83.40 ± 20.41 [a] | 94.37 ± 1.73 [b] | 198.29 ± 21.88 [a] |
| | Coarse | 16.05 ± 0.36 [g-j] | 39.38 ± 5.25 [b-e] | 69.75 ± 0.65 [c-e] | 125.96 ± 5.58 [d-f] |

Results are expressed as the mean ± S.D. in μg/g sample. Different letters per column indicate significant differences ($p < 0.05$) by Tukey-Kramer's test. **n. d.:** Not detected.

**Table S7**. Pearson and Spearman correlation analyses of phytochemical composition and eGI of popcorn samples.

| Variable 1 | Variable 2 | Pearson *r* | p | Spearman ϱ | p |
|---|---|---|---|---|---|
| IDF | eGI | -0.0765 | 0.8855 | -0.0857 | 0.8717 |
| SDF | eGI | -0.3710 | 0.4690 | -0.4282 | 0.3965 |
| Total Free Phenolics | eGI | 0.7630 | 0.0776 | 0.7714 | 0.0724 |
| Total Bound Phenolics | eGI | 0.9476 | 0.0040* | 0.8286 | 0.0416* |
| Total Phytosterol | eGI | -0.8630 | 0.0269* | -0.2571 | 0.6228 |
| ABTS | eGI | -0.0076 | 0.9886 | 0.1429 | 0.7872 |
| DPPH | eGI | -0.1430 | 0.7869 | -01429 | 0.7872 |

Values represent correlation coefficients calculated using hybrid means (n = 6). Pearson's *r* and Spearman's ϱ are reported together with their corresponding *p*-values. The asterisks indicate significant correlations at $p<0.05$.

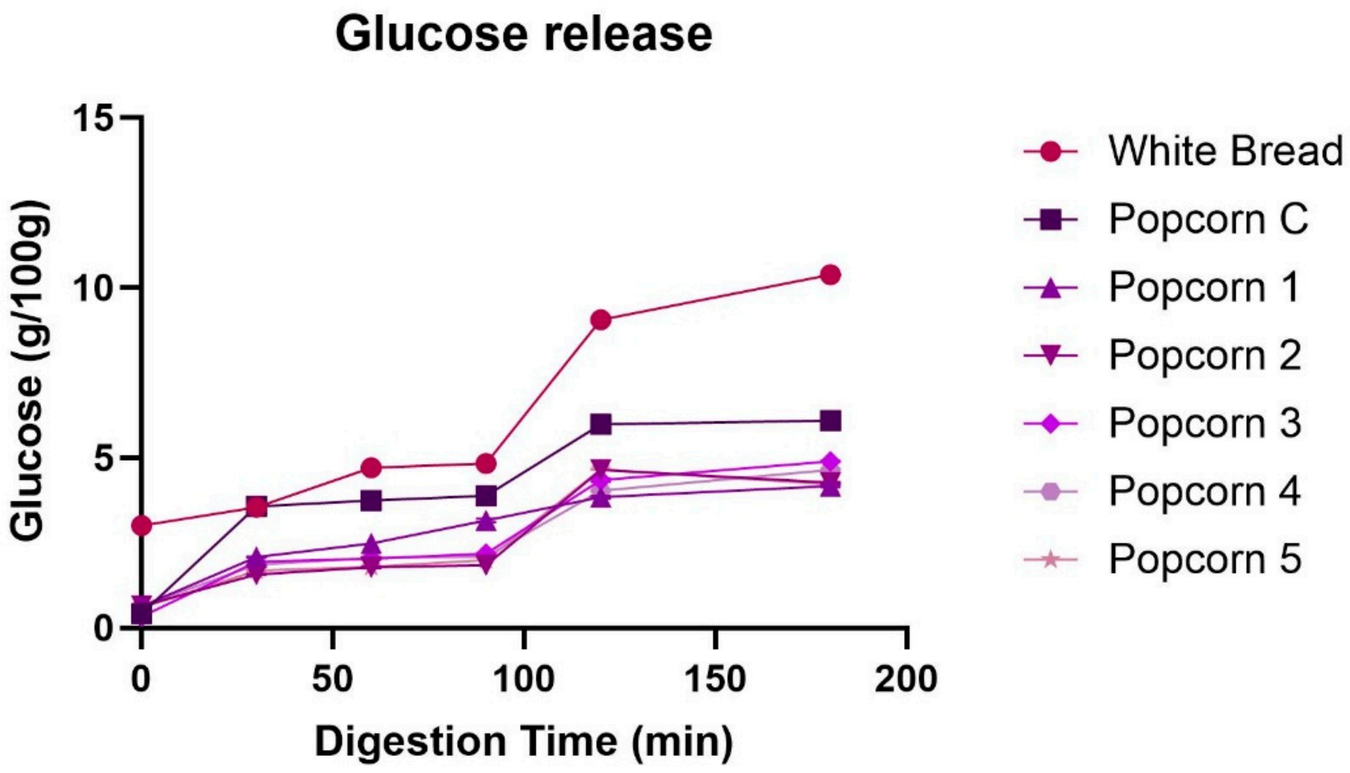


**Figure S1.** Release of glucose over time of different popcorn hybrid samples.

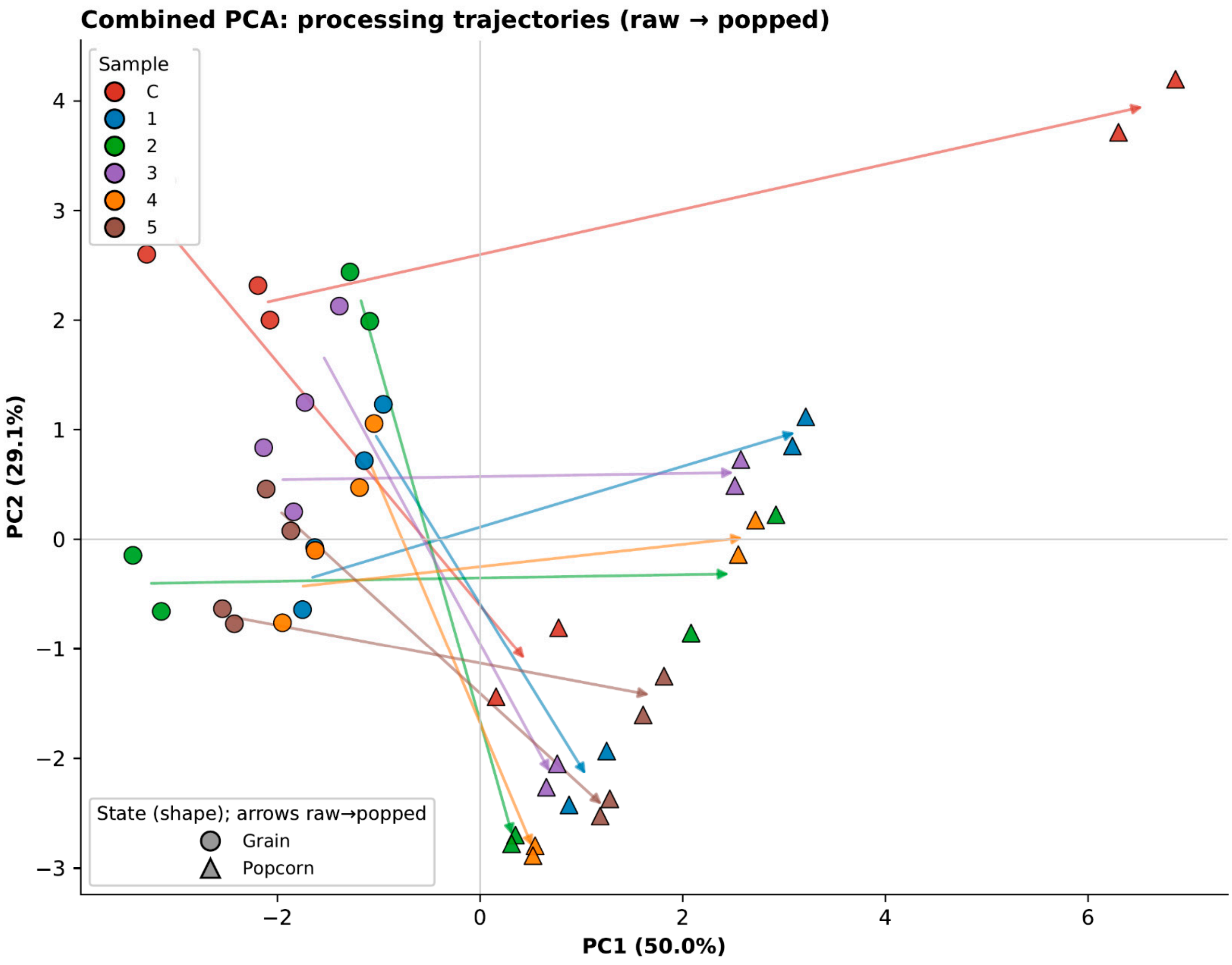


**Figure S2.** Combined PCA of the harmonized phenolic profile of raw grain (circles) and popped product (triangles); arrows connect the raw and popped centroids of each genotype × fraction combination, depicting the processing trajectory.
